# Extreme heat reduces and reshapes urban mobility

**DOI:** 10.1093/pnasnexus/pgag078

**Published:** 2026-04-14

**Authors:** Andrew Renninger, Carmen Cabrera

**Affiliations:** Center for Advanced Spatial Analysis, University College London, London W1T 4TJ, United Kingdom; Geographic Data Science Lab, University of Liverpool, Liverpool L69 7ZX, United Kingdom

**Keywords:** human mobility, cities, extreme heat

## Abstract

Extreme heat is a problem in Southern European countries and cities, aggravated by rising temperatures and aging populations. Research on mobility during extreme heat remains limited to small samples and geographic contexts, leaving gaps in our systematic understanding of how populations adjust their day-to-day mobility patterns and how these adaptations vary across social groups. Here, we use mobile phone data from passive and active mobile network connections covering 13 million individuals in Spain (27% of the population) to examine extreme heat’s impact on mobility at scale. We stratify by age, gender, economic class, and type of activity. Our findings show mobility falls by as much as 10% on hot days generally and 20% on hot afternoons specifically, when temperatures peak. Further differences emerge on hot days. Older adults cut travel to work and other activities, while those earning less are less able to avoid work; social mixing declines and spatial structure changes as activity falls in city centers. These disruptions have implications for urban economies, as curbed activity and interaction could threaten the dynamism of cities as hubs of social and economic exchange.

Significance statement While research documents behavioral adaptations to heat, we lack systematic, empirical understanding of how these adaptations change daily urban mobility patterns. Here, we combine location-based digital trace mobility data from mobile phones with high-resolution climate data, to provide evidence that heat not only reduces activity and restructures mobility, but that it exerts variable pressures on different groups, creating vulnerabilities and potentially amplifying social inequalities. Our work has implications for creating sustainable and livable cities. Specifically, our methodology and findings help facilitate the monitoring of mobility responses to extreme heat and have the potential to inform the implementation of measures to mitigate heat-related disruptions, such as urban greening and other ambient cooling strategies.

## Introduction

Extreme heat poses a serious threat to lives, livelihoods, and the economy (1, 2). Rising temperatures have been linked to increased hospital admissions (3, 4) and increased mortality rates (5, 6). Extreme heat reduces productivity in both manufacturing (7) and agriculture (8), and slows economic growth (9, 10). These challenges have been compounded by the growing intensity and duration of heat waves over the past century (11, 12).

The effects of extreme heat are uneven across populations, as some groups are more vulnerable or exposed than others (1, 13). For example, evidence suggest that the elderly are disproportionately affected by extreme temperatures (14, 15) due to increased vulnerability, often associated with chronic conditions such as diabetes (16, 17), which heighten their risk during heat events (18). Workers in physical labor (19), such as construction and agriculture (20), face significant risks due to prolonged exposure to extreme temperatures. Socioeconomic status determines adaptive capacity, as wealthier households turn on air conditioning at lower temperatures than poorer households (21). The widespread adoption of air conditioning has reduced heat-related mortality, highlighting the importance of wealth in adapting to climate stress (22, 23).

Individuals respond to hot weather in multiple ways. For example, time-use studies suggest that individuals reduce outdoor activities and shift toward indoor spaces during heat waves (24, 25), yet such shifts may be constrained by income, occupation, or urban design (25). Changes in mode of transport represent important means of adaptation (26–29). Cities see more cyclists and pedestrians on warm days than on hot and humid days (28, 30). Extreme heat can result in decreased productivity (31) at the workplace and more employees missing work. Social and economic incentives may influence decisions, with the risk of heat stroke heightened for the military and during athletic competitions (32). Further, shocks to infrastructure may either encourage or discourage adaptation, with railways, roadways, and energy grids experiencing greater strain during heat waves (33).

Critical questions remain about our empirical understanding of how heat shapes human mobility across different populations (28). Existing work is generally limited in scope and scale due to constraints in the availability of data. However, the recent raise of location-based digital trace data presents an unprecedented opportunity to study these behavioral dynamics systematically (34). These data allow us to monitor individual movement patterns at small spatial and temporal scales, capturing changes in mobility in response to extreme weather events, from fires to floods (35, 36), and the variability of these patterns across population groups (37).

The Southern European context provides the setting for the current study, characterized by historically temperate climate but a sharp rise in extreme heat events (38). Compared to other regions, populations show increased vulnerability to heat (39), and contrary to what evidence for adaptation would suggest (40), recent heat waves have been equally fatal as those in prior decades (41, 42). Aging population, with growing rates of chronic conditions like diabetes (16, 17), exacerbates the risk from heat, as both age (14, 15) and chronic illness (18) are associated with greater risk from extreme heat. The low adoption of air conditioning compared to regions like the United States (43) also creates risks and forces populations to rely on behavioral adaptations to cope with rising temperatures.

Here, we focus on Spain. Projections for the next 50 years suggest that Southern Europe, including Spain, will experience a combination of rising temperatures, increased drought frequency, and aging infrastructure (44). Heat waves in this region are expected to become more intense and spatially expansive (45). These challenges highlight the urgent need for studies that can inform adaptation strategies tailored to Spanish cities and populations.

The contribution of this work is to provide quantitative evidence of changes in daily mobility patterns in Spain in response to extreme heat, and to identify systematic variations in these changes according to the type of activity, as well as demographic and socioeconomic disparities. To this end, we combine large-scale mobility data collected in 2022 and 2023 with high-resolution estimates of thermal comfort. Our findings indicate that mobility related to nonroutine activities declines more sharply than for routine activities in response to extreme heat. Older populations reduce their number of trips more than younger groups; and less affluent groups show smaller changes in mobility compared to wealthier populations. Critically, we document how extreme heat systematically reorganizes urban network structures and reduces socioeconomic mixing, threatening the role of cities as engines of productivity and innovation (46). Cities thrive on interactions (47, 48) and systematic reductions in these interactions could suppress the benefits of agglomeration.

## Results

To understand the effect of heat on activity, we start by linking data on thermal comfort with data on daily mobility. We then apply several modeling techniques to detect statistically significant changes in mobility behavior across various population groups.

We use data provided by the Spanish Ministry for Transport and Sustainable Mobility (49), which contains records for the movements of ∼13 million individuals, or ∼27% of the population (see Supplementary Sections 1 and 7 for more information on descriptive statistics). Trips are logged from both active events like texts and calls as well as passive events in the form of probes from the network operator, allowing high temporal and spatial resolution. Because they come from network operators rather than applications, these data have comparably less bias than data from aggregators of GPS location data and they are validated and balanced with surveys and administrative statistics to ensure quality and reliability (see Methods for more details).

**Figure 1:**
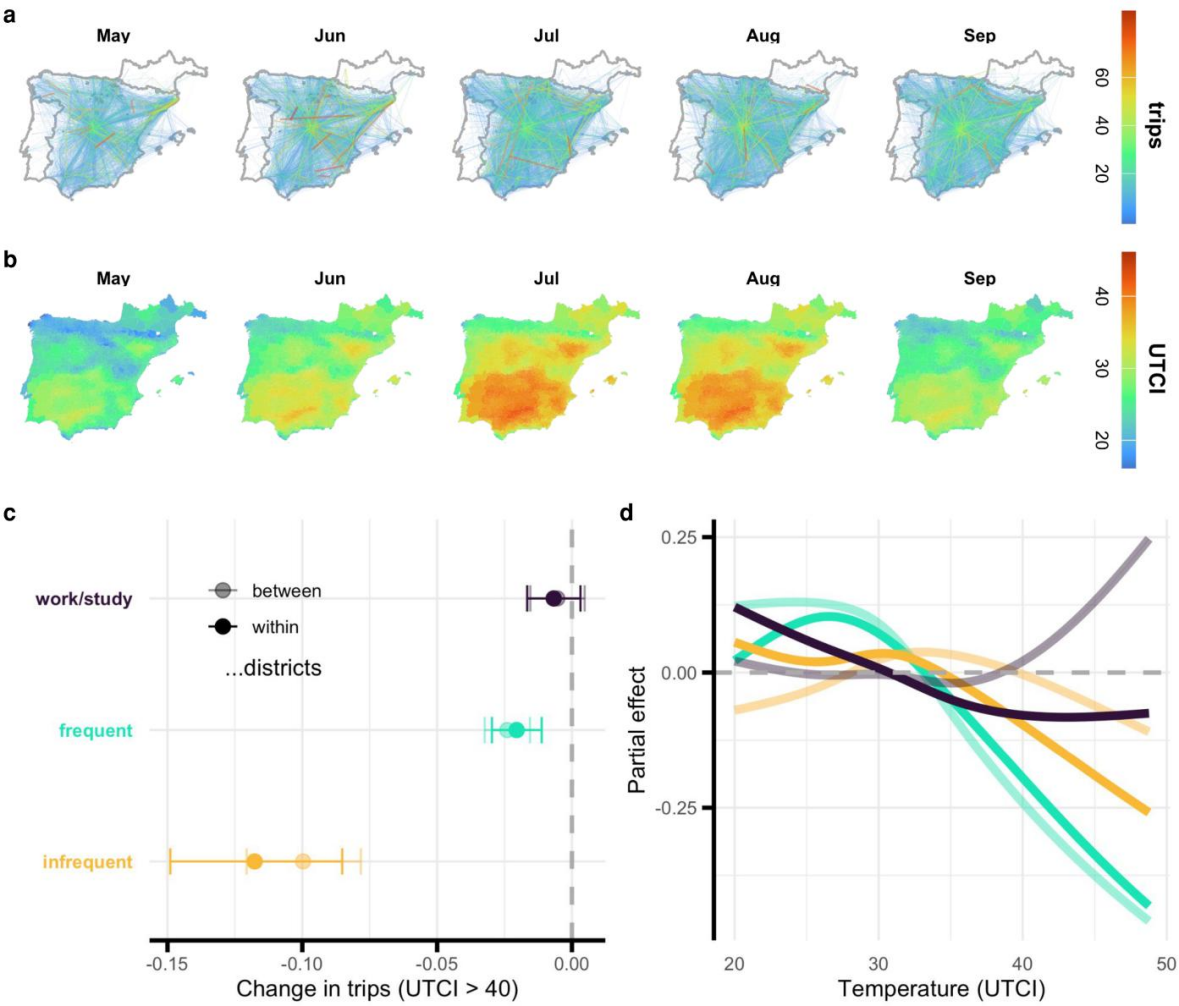
The effect of extreme heat on activity. a) Mobility networks in Spain across months in 2022 and 2023; the data represent 30% of the population, so the networks have good coverage, including rural and urban areas, although the network is dominated by cities like Madrid. b) Mean temperatures per month in the same period, with heat peaking in July and August. c) Estimates from a TWFE model, controlling for district and date, on the effect of a UTCI above 40°C, showing that frequent and infrequent activities fall while trips to work or school hold steady. Error bars denote 95% CI. d) When temperature is considered as a continuous variable rather than a simple binary condition, we find that mobility may increase for both frequent and infrequent activities when temperatures are below 40°C. For these trips, at very high temperatures above 40°C, mobility consistently decreases. For work and study, within- and between-district components diverge at high temperatures, suggesting resilience for longer journeys.

The data are made available to us as aggregated flows within and between 3,599 districts, including mainland Spain and the Balearic Islands. We do not have access to individual trajectories. Flows are pre-stratified by activity type, trip distance, age band, gender, and economic class. According to the Ministry, an activity is classified as “frequent” if an individual visits a location more than once within a 2-week period, and “infrequent” otherwise. Among the activity types, the “trabajo/estudio” (“work/study”) category refers to trips to locations associated with full-time employment or study. It is worth noting that although the overall share of individuals aged 65–100 in formal employment is relatively low, it is not negligible (50), and we still observe participation in the work/study category among this group. Moreover, given that the methodological documentation does not specify the criteria or algorithms used to assign trip purposes, it is plausible that some recurring mobility patterns, such as caregiving or other routine nonemployment activities, may also be captured under the “work/study” label. Figure 1A shows the networks for each month, demonstrating its strong coverage in both urban and rural areas, and we show time series and validation in Figs. S1–S3.

We measure daily variations in experienced heat using ERA5-HEAT data (51), which gives the Universal Thermal Climate Index (UTCI). This metric reflects perceived ambient conditions by incorporating temperature, humidity, wind, and solar radiation into a standardized formula. Focusing specifically on the impact of extreme heat, our analysis is restricted to the summer months, defined as May through September, for the years 2022 and 2023. Figure 1b shows the mean UTCI across months, with the strongest temperatures in July and August. Spain experienced significant heat waves in both periods, with 2022 seeing waves from June through August, while 2023 had milder conditions with record highs in August.

### Heat drives a sharp decline in travel for discretionary activities, with limited impact on work or study

We use a two-way fixed effects (TWFE) model to estimate the causal effect of temperature on activity by exploiting variation in temperature while controlling for both time-invariant attributes and spatially uniform shocks through district and date fixed effects (see Supplementary Section 2 for more information. Shown in Fig. 1c (and reported in Table S2), infrequent trips fall around 10% and trips to work or school see little change, with frequent trips falling almost 2.5%. Placebo tests, where we shuffle UTCI either by date or district, in Fig. S5, show no effect for permuted UTCI on activity, indicating that the results are not spurious.

To identify the form of the relationship between temperature and activity, we turn to a generalized additive model (GAM), which fits smooth functions to capture nonlinear relationships in data and in doing so extract effects across different temperatures. Our specification is similar to above, but we fit a cubic spline by day-of-year to model the seasonality, and include day-of-week and holiday terms to account for variations across days. Showing this continuous relationship between heat and activity in Fig. 1d, we see that higher temperatures result in lower activity. Although this model disagrees with the TWFE in which activities are most responsive to heat, the GAM shows that there is a temperature at which activity in both categories peaks. In Fig. S4, we show these patterns are consistent when we limit our sample to large cities or small cities, and when we hold out 2022 or 2023. This suggests that as climate patterns shift over time, some seasons and places will see visits and trips increase as warmer weather generates activity while others will see them decrease as hotter weather destroys activity.

In the TWFE, work and study coefficients are small and statistically indistinguishable from zero across temperature bins. Further, infrequent trips within a district—those more plausibly traversed on foot—fall more than those between districts. In the GAM, we see a functional form consistent with peaking activity at mild temperatures, with the exception of work and study, which remain comparatively flat. The GAM also shows some divergence between within- and between-district flows at high temperatures; between-district trips show smaller declines (and in some cases modest increases), which we interpret as resilience. One possible explanation is that longer trips are more likely to involve motorized transport (or other air-conditioned transport) and thus be less exposed to heat. Next, we leverage the rich demographic and geographic attributes to examine how these effects vary across different populations and contexts.

### Extreme heat affects mobility most for the elderly and poor, with no significant gender differences

In addition to activities, we disaggregate according to various demographic attributes, including age, gender and income. Our preferred specification is the TWFE which, under certain assumptions (see Methods section), allows us to estimate a causal effect of extreme heat on mobility. Given the functional form indicated by the GAM, here we use temperature bands rather than a binary indicator of extreme heat. Our estimates are both statistically and practically significant, showing reductions across all classes of activity. The patterns we see in this section vary systematically between activities and monotonically across temperatures, lending confidence to the relationship we see across point estimates: higher temperatures mean lower mobility. Note that the results that follow are robust to excluding August from the estimation, which we show in Tables S3–S6, when many in Spain take vacation (52, 53).

**Figure 2:**
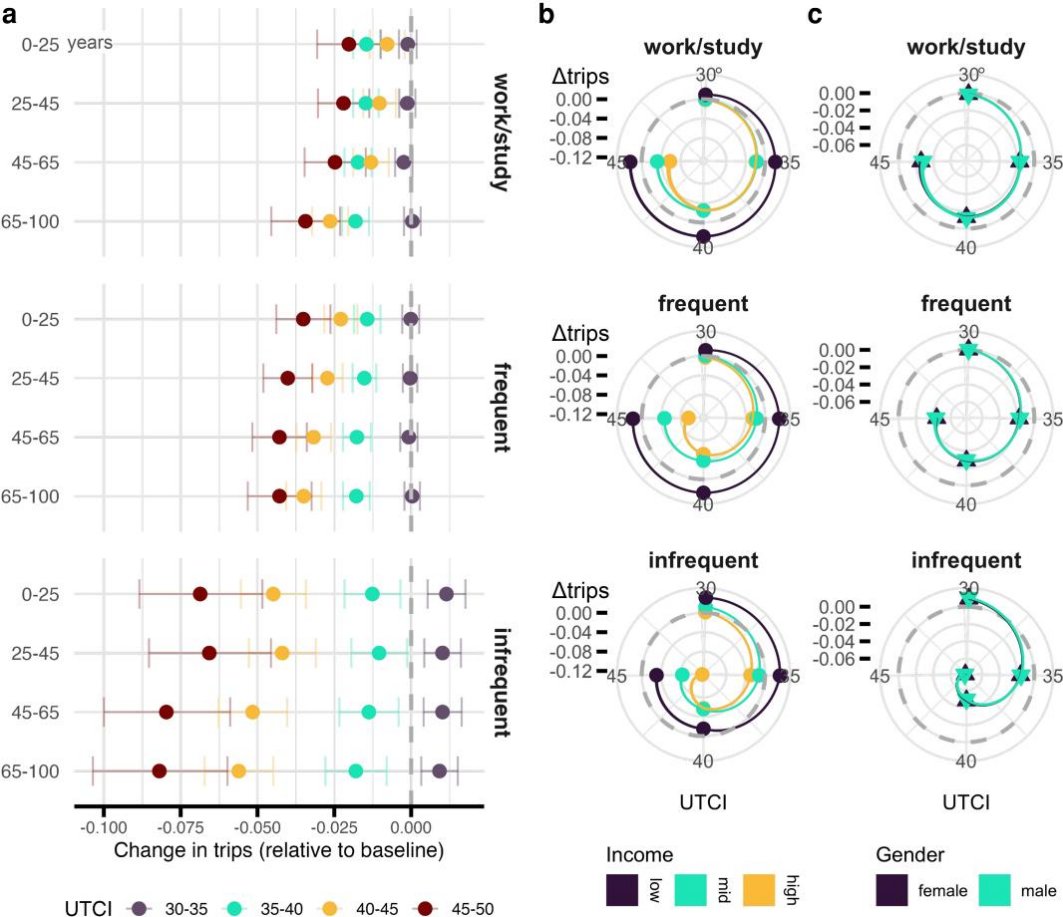
The effect of extreme heat on different groups. Effects are relative to the omitted 25–30°C UTCI baseline bin and are semielasticities (log points), so −0.10≈−10%. a) Disaggregating by age, we see that as temperatures increase and the regularity of the activity decreases, trips decline, but more for the oldest than the youngest people. We also note that warm-not-hot temperatures generate infrequent activities, people may expand their repertoire of activities during clement weather. Error bars denote 95% CI. b) Change in effect size as UTCI increases for different income brackets, showing that the poor less sensitive to high temperatures than the wealthy, which may be tied to work-from-home, although all groups reduce infrequent activities. This suggests that discretionary mobility changes but obligations like work do not. c) We see no differences by gender, with activity falling identically as UTCI rises for both males and females despite aggregate differences in mobility between the genders.

In Fig. 2a, we see a gradient, with higher temperatures corresponding to stronger declines in activity across all age groups. Looking at how different ages respond to extreme heat, our results are clear: mobility for the young is the least affected by high temperatures and the impact becomes larger as age increases. For the oldest group, a given day with heat index above 45°C corresponds to an 8% decline in infrequent activity, a 4% decline in frequent activity, and even a 3% change in work or study. Those in middle age visit work 2.5% less on the hottest days. Because we are using data from 2022 and 2023, we note that these effects do not necessarily mean a reduction in work, because the foregone travel could be to work from home. Yet, this change in behavior would still have implications for cities as Spain warms over time: if the elderly are *missing* work, there is a direct economic cost to the workers, but if many are simply working from home, then the economic burden falls on the shops and restaurants that rely on business from commuters.

This is consistent with the fact that heat poses a greater risk to older populations than it does to younger ones (15). In relative terms, the oldest are most affected by extreme heat but because they constitute a larger and more active population, the greatest decline in absolute terms comes from the middle-aged population, while younger populations are least impacted in both absolute and relative terms.

There are many plausible channels by which income and heat could interact but here we propose two: the wealthy might be more capable of coping with extreme heat, via air conditioned homes and cars, and thus remain unaffected; the poor might be less able to afford missing work. White collar jobs allow at least some remote work and many blue collar jobs do not. Shown in Fig. 2b, our results indicate that the poor cannot afford, or are not able, to miss work. Individuals from households in the lowest income bracket are unaffected by high temperatures while those from households in the highest income bracket reduce travel across all classes of activity.

Supporting the hypothesis that work compels the least affluent group to stay active, we see that this group still curbs infrequent activities while holding steady trips for work or study as well as for frequent activities even when the heat index surpasses 45°C. These frequent activities could be attached to daily or weekly routines like lunch breaks, or taking children to school and therefore co-occur with work. For the wealthiest group, all three classes of activity fall at that level of discomfort—by as much as 10% and 15% for frequent and infrequent activities, respectively.

With large differences in labor force participation between men and women (54), and differences in both unpaid work and care work between men and women (55), we might expect routines to vary enough to see variation in responses to heat. Yet once we stratify on the type of activity in Fig. 2c, we observe no gender differences in mobility. (Descriptive statistics and the distribution of trips by age, income, and sex are reported in Table S1 and Figs. S6–S8, but, we note here no detectable differences between genders in the distributions).

### Larger drops in the afternoon and on short trips that may involve active travel

**Figure 3:**
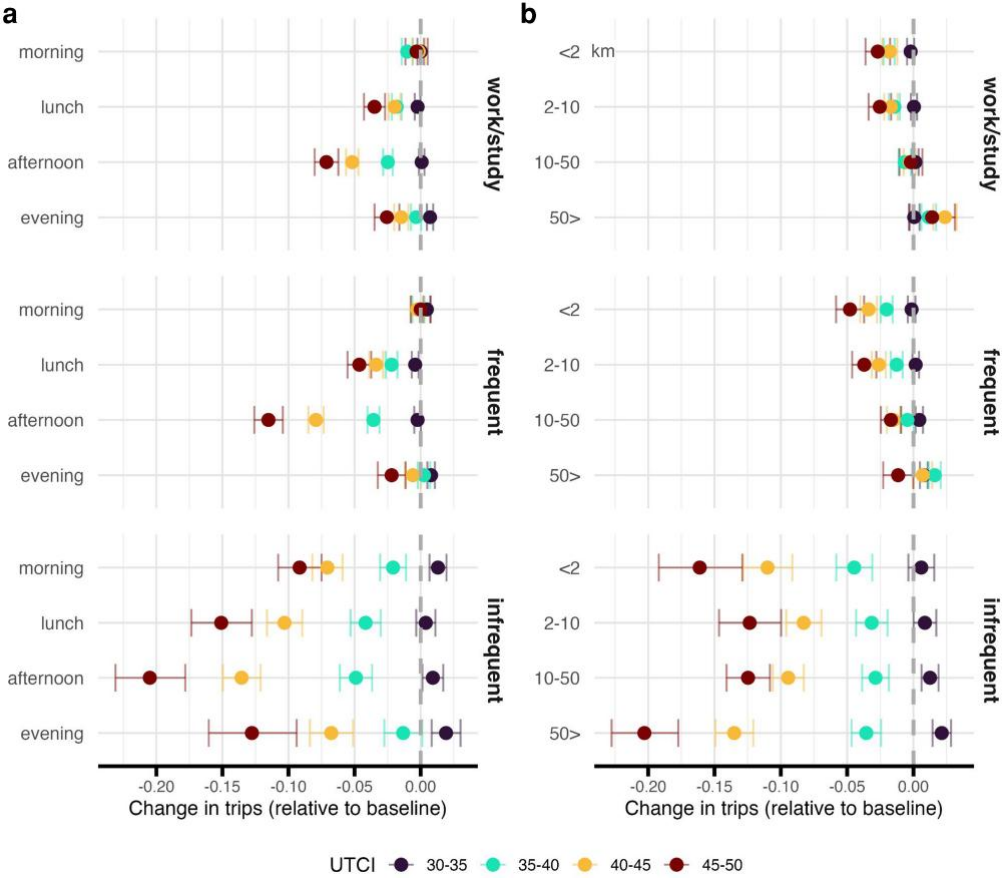
Time and distance. Effects are relative to the omitted 25–30°C UTCI baseline bin and are semielasticities (log points), so −0.10≈−10%. Error bars denote 95% CI. a) We see patterns consistent with our earlier findings, wherein trips to frequently visited locations fall more than trips to work or school, and trips to infrequently visited locations fall most, but we also see that this effect is pronounced in the afternoon when temperatures are crest. We do not see evidence of substitution to the morning or evening before or after temperatures peak, as those trips still decline on hot days. b) Generally, longer trips (distance in km in the *y*-axis) are most resilient to extreme heat than shorter trips, and cars may play a role in this difference as shorter trips are more likely to be taken on foot. Mobility related to infrequent activity sees strong changes for both long and short trips.

Because heat is variable throughout the day, starting off cooler in the morning, heating up in the afternoon and cooling later in the evening, we test whether or not people respond this progression. Figure 3a shows that visits to all classes of activity fall more in the afternoon on hot days than they do in the morning, by as much as 20% for infrequent activities on the hottest days of the year. Yet even frequent activities, which may be coupled with work or study, fall by more than 10%. Taken together, this also lends credibility to our earlier estimates because it shows that mobility responds not just to hot days but the hottest part of the day, which would be less likely if we were observing a spurious effect. It also suggests that even when people go to work on a hot day, they might do fewer other activities during the those afternoons.

In Fig. 3b, we explore effects across different journey lengths. Our data do not allow us to interrogate why people might be avoiding certain kinds of travel, but in our models we see that the largest reduction in activity that comes from trips that span <2 km, which are less likely to involve a car. This agrees with literature showing that cycling and walking are most impacted by hot days (28). Again we see tight coupling between mobility for work or study and for frequent activities, suggesting that certain activities might go hand-in-hand with a routine that includes both professional obligations and personal needs. Although long trips are generally the least affected by high temperatures, they experience the largest declines when they involve infrequent activities, specifically when UTCI exceeds 40°C.

### Reduced social mixing as temperature rises

**Figure 4:**
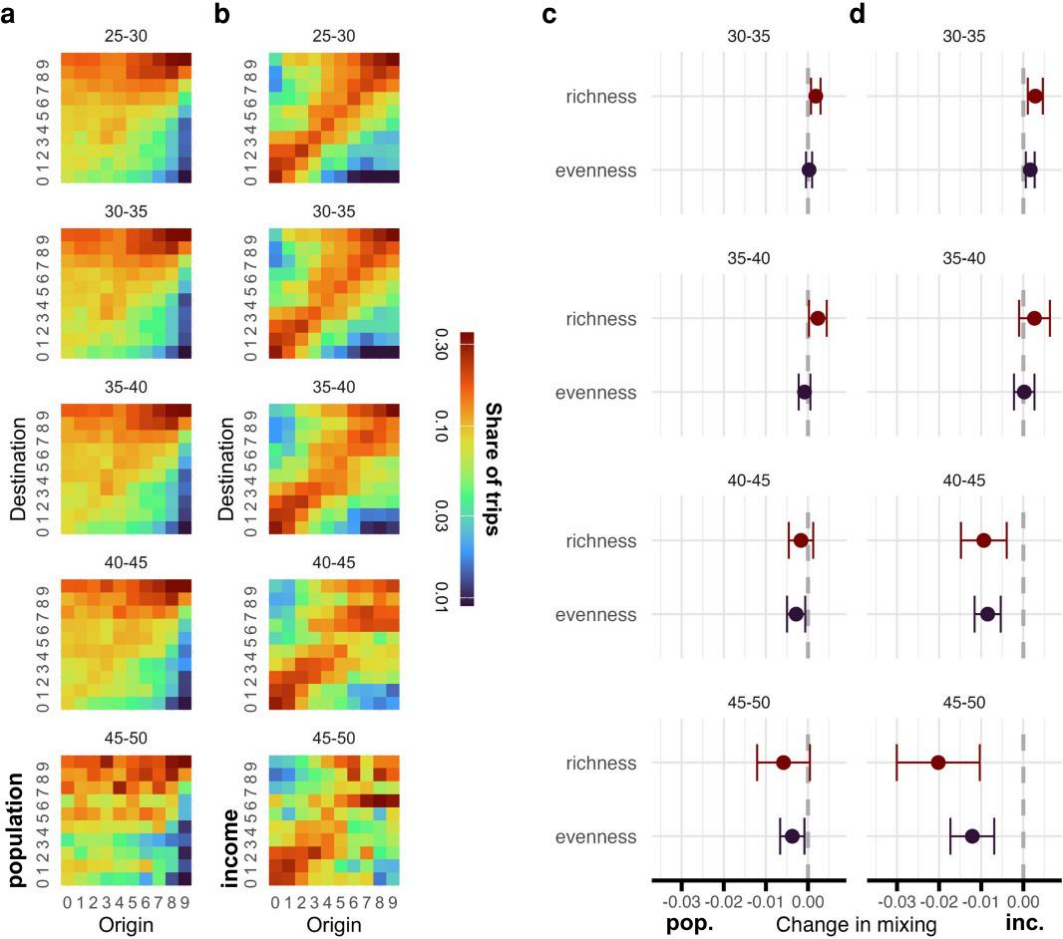
Mobility patterns and heat. a) Changes in flows between districts ordered by population decile, showing that at normal temperatures most flows go from low population to high population areas, or stay within high population districts, but this relationship breaks down during the hottest temperatures and more trips flow between what are suburban densities. b) Changes in flows between districts ordered by median income decile, showing that there is a subtle but consistent bias wherein people from middle income districts tend to visit upper income districts, and this relationship also fades at higher temperatures—although trips continue to flow between lower income districts. c) The results of a more controlled test, using a TWFE to predict the change in richness and evenness of visitors to a destination according to origin population, and d) according to origin income; we see a stronger decline income diversity and the same gradient we see with total activity, with stronger temperatures generating stronger effects. For c and d), effects are relative to the omitted 25–30°C UTCI baseline bin and are semielasticities (log points), so −0.10≈−10%; error bars denote 95% CI.

We also document significant changes to the structure of the mobility network as temperature changes, which may have implications for how urban areas function and how social groups mix. Figure 4a and b shows how trips flow to and from districts with different populations and different incomes (in deciles), respectively. Generally, trips flow from less populous to more populous areas (urban bias), and from lower income to higher income (wealth bias). These patterns are marked by the higher values in the upper triangles of the matrices in Fig. 4a and b. We stratify on UTCI to show how these mixing profiles vary under different climatic conditions and plot results by row according to different UTCI bands. When it becomes hotter, these twin biases attenuate. More trips occur within middle quantiles, and in particular few trips flow from middle income to high income. Agreeing with our earlier results showing limited change amongst the poorest, flows within and between low income districts holds constant. (Note that although districts are large units, with ∼8,000 residents, the ratio of *between*-district to *within*-district flows is 3:1, so while many needs are met within each district there is substantial potential for mixing).

Building on these descriptive results, we introduce another TWFE design to relate mixing with temperature, using metrics borrowed from ecology (56): *richness*, defined as the raw number of income or population groups who visit, and *evenness*, measured using the Shannon entropy of visitor distribution across these population or income groups. The new metrics, richness and evenness, are used as the dependent variables in our TWFE model. Looking at mixing between low and high population districts in Fig. 4c, we see less of a change than what is visible in the matrices, although there is a slight reduction in mixing between rural, exurban, suburban, and urban classes at the highest extremes. Looking at mixing by economic class in Fig. 4d, however, richness falls by ∼2% and evenness falls by ∼1.2% on very hot days. Taken together, this suggests that much of the change in mobility that we see in the matrices is attributable to seasonal variations, yet it also shows a direct effect from heat on mixing. As we move from lower to higher temperatures, we see a consistent progression; we also see evidence for mixing on mild days, fitting with earlier indications that some temperatures are conducive to new activities. Taken together, these findings suggest that extreme heat not only reduces overall mobility but systematically alters the network structures that determine core–periphery interaction and socioeconomic mixing.

**Figure 5:**
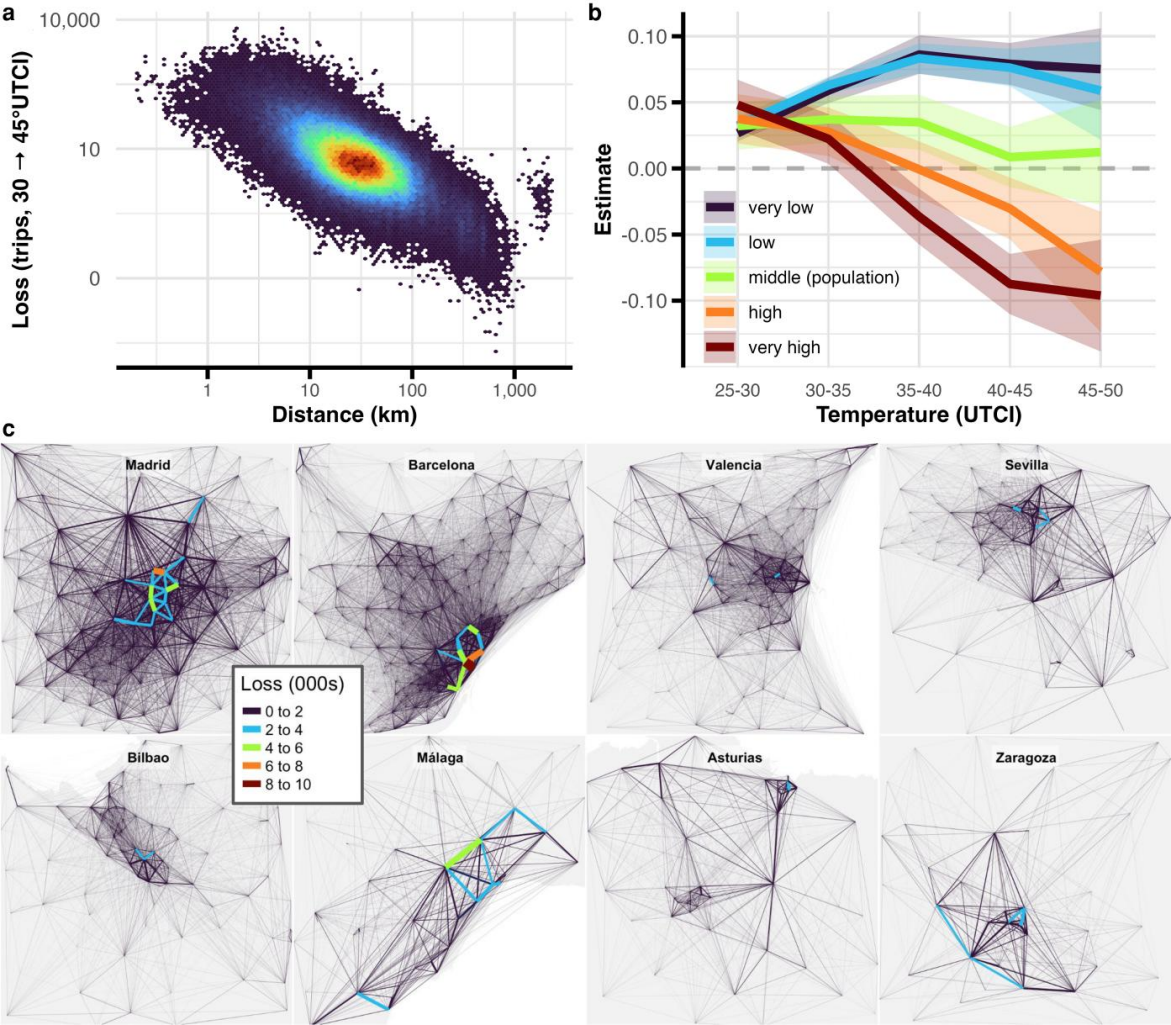
Urban impacts. a) We build a network of trips between districts and show the relationship between the loss of trips between districts and the distance between them, when temperatures rise from 35 to 40°C, according to a gravity model with temperature, finding a clear negative slope. b) In order to model how different parts the rural–urban gradient respond to heat, we add population quantiles to that gravity model, and see that more populous areas see larger reductions in trips. c) Building out the networks from this gravity model, we can see that edges in the core see the largest declines in flows as temperatures rise from 35→40°C, while edges in the periphery are preserved (ribbons denote 95% CI).

We model changes to urban structure formally using a gravity model that considers population at origin and destination, along with distance between districts and the temperature on the day. The gravity coefficients, shown in Table S8, confirm the earlier models but gives us the ability to observe changes on the network. In Fig. 5a and b, we show the components of that model: shorter edges see flows decline more on hot days that longer edges, and flows between populous areas fall most. Rural areas see mobility taper off from the optimum—at ∼35°C but the net effect relative to cold weather is still positive. We see the consequences of this in Fig. 5c, in that losses on hot days tend to be concentrated in the urban core rather than the periphery. Generally, in this network analysis, we see that social life in cities will be disrupted if extreme heat worsens without adaptation, with less mixing and less activity in city centers—a development which could threaten the economic and social advantages of cities.

To quantify possible changes going forward in time, we use state-of-the-art climate forecasts to estimate changes to activity as the climate changes in Supplementary Section 9.

## Discussion

We find that mobility responds to heat in ways that are consistent with expectations rooted in the literature on extreme heat and health. First, daily movement patterns for the elderly are most affected by extreme heat; second, afternoons see the greatest decline in mobility levels across population groups, as temperature crests. We find that those older than 65 are more likely to reduce activity in response to high temperatures, and that their reductions are greater at higher temperatures. Mirroring the daily temperature dynamics, we see that on hot days activity falls most during the early and late afternoon, and least in the mornings and evenings. Again, our results show that as temperature increases, so does the effect size in our model estimations. These changes impact the spatial structure and social mixing in cities.

We document opposing effects from extreme heat amongst another vulnerable group. While the oldest reduce discretionary activities and skip travel to work, the poorest still commute and generally, reduce activities less. This means that the group most at risk, because age magnifies the threat of heat, is responding according to that risk, but it also suggests that the poor are least able to compensate for extreme heat by foregoing work and travel. This reveals important economic constraints that may influence mortality and morbidity.

Lending confidence to our estimates and findings here is the consistency with which our models behave: higher temperatures correspond with larger effects in all of our specifications. Although these findings appear intuitive, our study is the first to document these changes accounting for trip and individual attributes. In doing so, it demonstrates the adaptive nature of human mobility in the presence of extreme heat: populations respond to high temperatures by changing routines and avoiding certain activities. Without more granular data, we cannot shed light on what all of these activities are specifically, but we do see patterns in the analysis we are able to make.

With an aging population and a warming world, our results suggest that policies to adapt to extreme heat are becoming increasing important for keeping Spain and possibly other Southern European countries active and productive in the coming decades. Yet many existing strategies to mitigate the worst health effects of extreme heat involve air conditioning (22), which is difficult to apply to activity. There are still strategies to mitigate extreme heat between buildings, like greening (57), and certain modes of travel can also be air conditioned, but a broad drive to move people to air conditioning could reduce interactions, and we find preliminary evidence of how this might occur here. The short trips that are more likely to be walked are also more likely to be avoided in extreme heat. On the hottest days, many also avoid travel to work, so businesses that depend on commuters for foot traffic might suffer. The neighborhoods at the hearts of cities lose the most activity. The very adaptations that make heat survivable might erode the subtle interactions that make cities engines of innovation (47, 48) and culture (46). Our findings suggest, while air conditioning and work-from-home may reduce the threat of extreme heat, cooling interventions across neighborhoods and cities, like greening and shading, or changing paving and building materials to reduce solar absorption, may preserve the interactions that define cities.

Although our results are consistent and intuitive, the mechanisms underlying the behavioral changes we see in the data during extreme heat are not clear. For example, in the current work, we do not prove a link between short trips and active travel, and although we know that frequent trips appear linked to work/study, which agrees with research on trip chaining (58), we do not determine which classes of amenities are most affected when people avoid travel to work. Our study is thus limited, providing strong evidence for adaptation while some of the more granular details are left to future research.

## Methods

We employ twin modeling strategies to understand the relationship between heat and mobility, the first to measure the causal effect and the second to estimate the functional form. Here, we explain the main specifications; for details on the variations of these models that we employ throughout the article, see Supplementary Section 2. Both assume the number of trips *T* terminating in district *i* at time *t* follow a Poisson distribution such that $T_{it}∼\text{Poisson}(\mu_{it})$. Our first approach uses a TWFE model:


$$\log\;(\mu_{it})=\beta ({\text{UTCI}}_{it}\times \text{activity})+\alpha_{i}+\gamma_{t},$$

where $\mu_{it}$ represents the expected number of trips, $\alpha_{i}$ represents district fixed effects controlling for characteristics of the *place*, while $\gamma_{t}$ captures date fixed effects accounting for patterns common across districts at a given *time*. UTCI is either binary (>40°C) or binned (5°C intervals from 20 to 50 °C). More information about the binned specification is found in Supplementary Section 2, but here we note that all values relative to 25--30°C. The interaction with activity type allows us to estimate differential temperature responses across activities. This specification leverages within-district variation in temperature after accounting for common temporal shocks, providing causal estimates under the assumption that temperature variation is as-good-as-random after controlling for location and time fixed effects. The district controls for spatial confounds and the date controls are important to adjust for temporal patterns, as Spain sees activity change considerably in August as many people make holidays during this month (52, 53). We cluster standard errors at the province level to account for spatial correlation in the error terms. While our method is robust to holidays and seasonal vacations because date fixed effects absorb common temporal shocks, we additionally re-estimate all models excluding August (Spain’s peak vacation month) and show side-by-side comparisons in Supplementary Section 4. We test for violations of the stable unit treatment value assumption (SUTVA) in Supplementary Table S7.

To explore and model the potential curvilinear relationship between heat and activity, we complement the TWFE analysis with a GAM:


$$\begin{array}{rl} \log\;(\mu_{it})\;= & \;f_{1}({\text{UTCI}}_{it})\times {\text{activity}}_{i}+\beta_{1}{\text{popularity}}_{i}+\beta_{2}\;{\text{province}}_{i}\\ & +f_{2}({\text{DoY}}_{t})+{\text{DoW}}_{t}+{\text{holiday}}_{t}, \end{array}$$

where $f_{1}(\cdot )$ represents a cubic regression spline with four knots, $f_{2}(\cdot )$ is a cubic spline for the day-of-year, capturing seasonality and drifts in the data, and we control for mean visitation (popularity) and geographic variation (province). We also add day-of-week and holiday fixed effects because, for example, weekends and holidays might have different levels of activity and this allows the intercept to vary on those days. While the TWFE isolates the causal effect, the GAM reveals the functional form of behavioral responses to temperature variation through its flexible smooth functions. The GAM’s strength lies in its ability to detect and convey nonlinear relationships without imposing a priori assumptions about the functional form, allowing us to identify potential threshold effects and complex response patterns in human mobility.

## Supplementary Material

pgag078_Supplementary_Data

## Data Availability

Mobility data are available from the Ministry for Transport which can be accessed at https://www.transportes.gob.es/ministerio/proyectos-singulares/estudios-de-movilidad-con-big-data/opendata-movilidad. ERA5 climate recordings and CMIP6 climate projections are accessible via Google Earth Engine. All code is available at https://github.com/asrenninger/movilidad.

## Preprints

This manuscript was posted as a preprint: https://doi.org/10.48550/arXiv.2501.03978.
