# Supplementary material for: Extreme heat reduces and reshapes urban mobility
Source: PNAS Nexus. 2026 Apr 14;5(4):pgag078. doi: 10.1093/pnasnexus/pgag078 (PMC13077673; doi:10.1093/pnasnexus/pgag078)
Supplement: pgag078_Supplementary_Data [file pgag078_supplementary_data.pdf]

Supplementary Material for

Extreme heat reduces and reshapes urban mobility

Andrew Renninger* and Carmen Cabrera

*Corresponding author: Andrew Renninger (E-mail: andrew.renninger.12@ucl.ac.uk)

Supplementary Notes

1 Data description **2**

2 Model description **7**

3 Model results **10**

4 Model comparisons **11**

5 Sensitivity analysis **15**

6 Placebo tests **16**

7 Consistency between groups **17**

8 Gravity model **20**

9 Projecting into the future **22**

1 Data description

We use daily origin-destination data provided by the Spanish Ministry for Transport [1], which integrates anonymised mobile phone records with demographic, land use, and transport network information to produce a mobility data product. This dataset captures trips over 500 meters within Spain and infers key travel characteristics, including origin and destination points, travel modes, and trip purposes—either work/study, frequent or infrequent locations for that device. The Ministry leverages state-of-the-art algorithms to transform raw mobile network data into structured and scalable matrices, offering high-resolution insights into mobility patterns across spatial and temporal scales. We gather this data for May-September 2022 and 2023 with the R programming language a the Spanish OD Data package [2]; by early 2022, national COVID-19 restrictions were lifted in Spain, with the exception of proof of vaccination for international travelers.

While many studies using GPS location data are only able to impute demographic attributes using administrative statistics [3, 4], often aggregated to large areal units that make the estimates crude, the data are stratified to allow for interrogation variations across demographic groups and trip purposes [5]. Activities are classified based on the Ministry of Transport's recurrent mobility analysis, which tracks origin-destination pairs over 2-week periods. Destinations visited more than once in this window are classified as frequent activities, while those visited only once are considered sporadic activities. Balance is achieved using official statistics from the national statistics agency to account for differences in age, income, and regional population distributions. This ensures that the dataset is representative of the broader population, minimizing biases associated with the uneven distribution of mobile phone users. The integration of demographic and geographic information also allows for the segmentation of mobility patterns by municipality, province, and other spatial units, providing a flexible foundation for granular analysis.

The Ministry for Transport employs rigorous quality controls to ensure the reliability of these data [6]. Anomalies in travel patterns are monitored through automated systems, which compare data against historical trends and predefined thresholds. Possible errors, such as geolocation inaccuracies or missing records, are flagged and investigated to ensure data integrity. The data are also validated with independent sources, such as FAMILITUR survey data, to confirm the consistency of observed trends with government statistics. Additionally, logical consistency checks, such as evaluating the symmetry of origin-destination flows, are conducted to ensure that the data align with expected behaviours. These efforts, combined with transparent methodological documentation, make this dataset an important resource for understanding mobility in Spain.

We link these data on mobility with an index of thermal comfort from ERA5-HEAT climate reanalysis data [7], provided by the Copernicus program. Universal Thermal Climate Index (UTCI) combines temperature, wind, radiation and humidity to measure not just how hot it is but how it feels—for example, if humidity limits evaporative cooling through perspiration. We use zonal statistics to compute the mean UTCI at 16:00 for each district on each day.

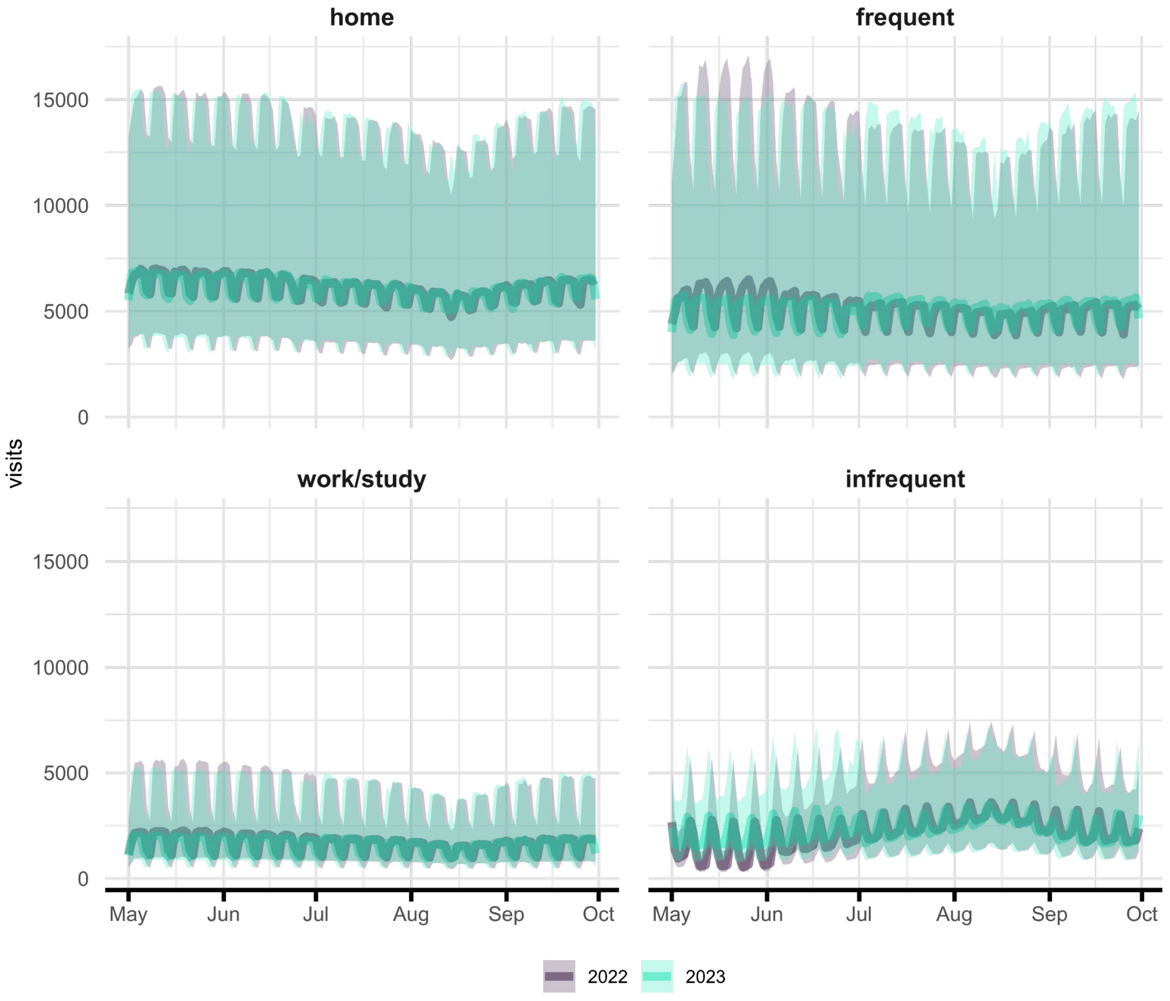

Figure S1: Trends in the data. Time series per activity over the two summers of our study, with interquartile range shaded. We see that work/study and frequent activites are more common during the weak and infrequent activities are more common on the weekend; there is a seasonal trend wherein infrequent activities rise is August while work/study and frequent activities fall. We also note that in May 2022, when school is not in session, frequent activities are higher and infrequent activities lower than in the same period during 2023—possibly due to a classification error. In light of these trends, we make adjustments to our model specifications to account for these weekday/weekend and seasonal variations.

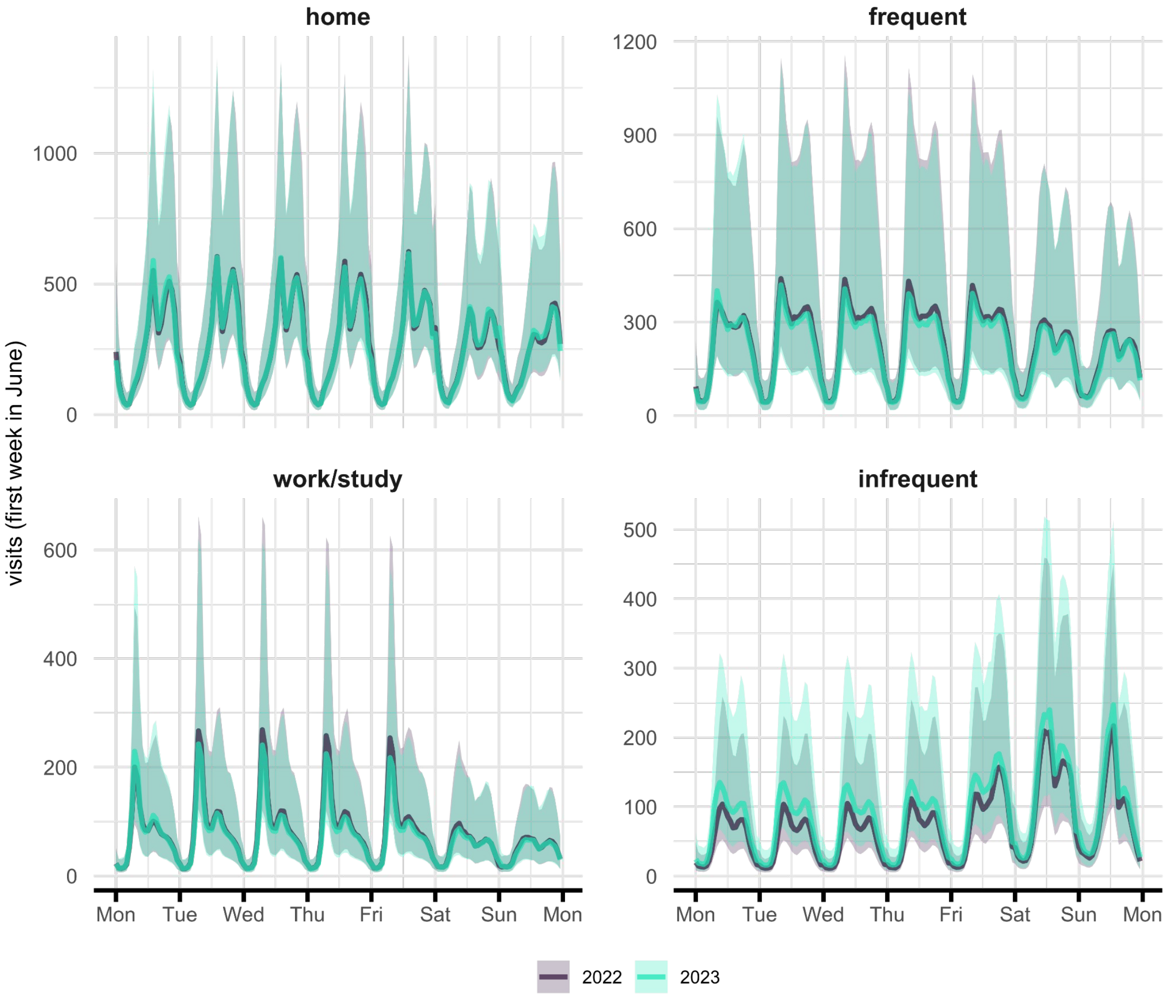

Figure S2: Weekly patterns. Time series per activity for the first week in June, with interquartile range shaded. We inverted home and work/study patterns, in line with commuting during the day, and we see that infrequent activities are more common on week days.

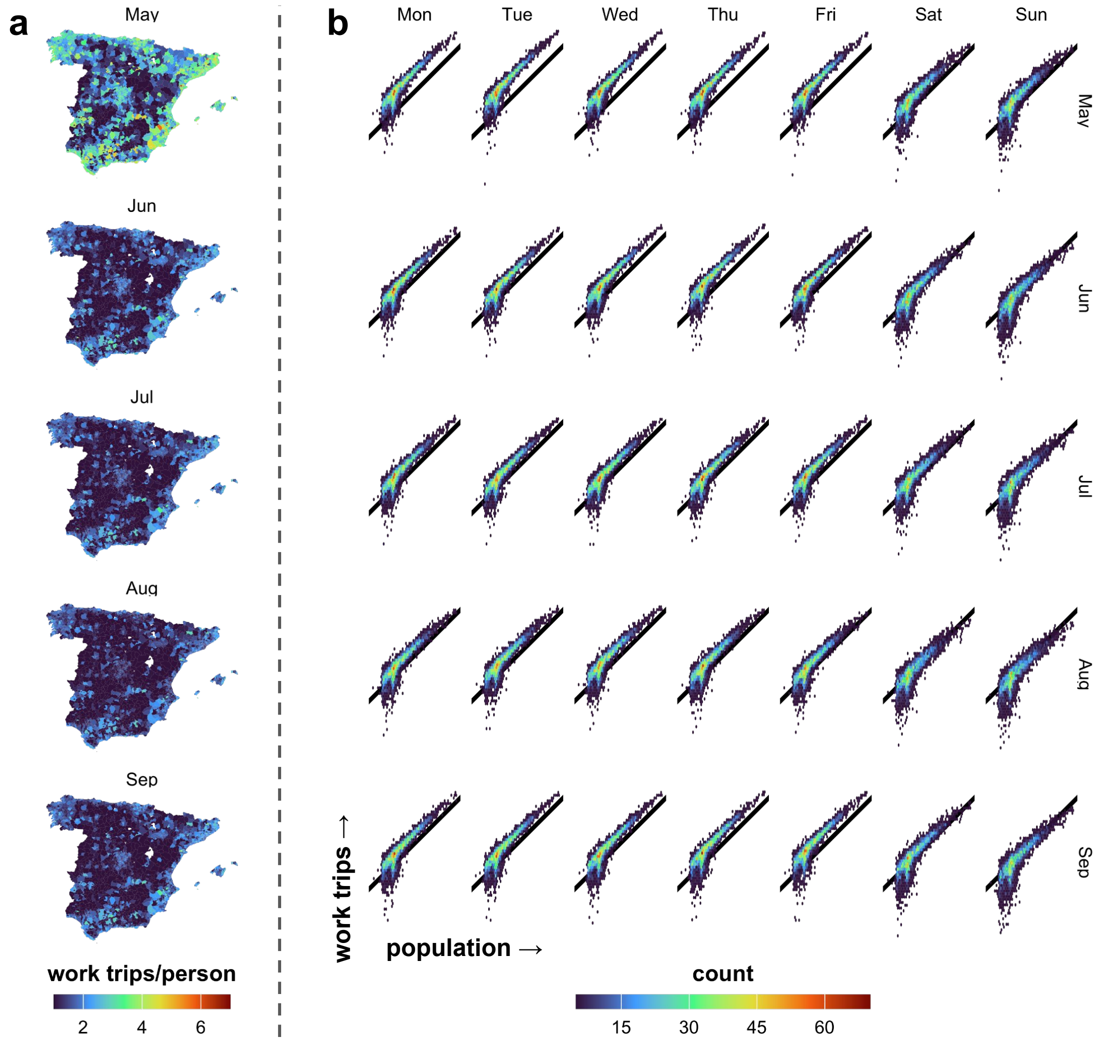

Figure S3: Mobility and population. A The ratio work trips per person in each district for each month. We see that again in May the balance is different than in the remaining months; and urban districts tend to have more work trips than rural ones. This is because school is still in session in may, and work/study includes both of these trips. **B** We document a strong correlation between work trips and population, again with the exception of may, which typically has more work trips per person than other months. The fit returns to that of other months on the weekends, when school is out, during May. We address this in the model specification, and we also perform sensitivity analysis including and excluding this period.

Table S1: Descriptive Statistics by Age, Income, and Sex

**Stratum**	**Activity**	*Trips* Total (billions)	Share (%)	*Observations* (millions)	*Districts*
Panel A: Age group					
	Work/Study	0.51	5.0	1.02	3599
0–25	Frequent activity	1.49	14.6	1.08	3599
	Infrequent activity	0.48	4.7	1.09	3599
	Work/Study	0.63	6.2	1.06	3599
25–45	Frequent activity	1.69	16.6	1.09	3599
	Infrequent activity	0.55	5.4	1.10	3599
	Work/Study	0.67	6.6	1.08	3599
45–65	Frequent activity	1.95	19.2	1.10	3599
	Infrequent activity	0.61	6.0	1.10	3599
	Work/Study	0.21	2.1	0.90	3594
65–100	Frequent activity	1.07	10.5	1.08	3599
	Infrequent activity	0.30	2.9	1.08	3599
Panel B: Household income					
	Work/Study	0.53	3.9	0.83	3586
< 10	Frequent activity	1.55	11.4	0.90	3599
	Infrequent activity	0.47	3.4	1.00	3599
	Work/Study	1.76	12.9	1.10	3599
10–15	Frequent activity	5.28	38.7	1.10	3599
	Infrequent activity	1.63	11.9	1.10	3599
	Work/Study	0.48	3.5	0.77	3500
> 15	Frequent activity	1.46	10.7	0.90	3599
	Infrequent activity	0.50	3.6	1.02	3599
Panel C: Sex					
	Work/Study	0.83	9.9	1.03	3599
Female	Frequent activity	2.70	32.2	1.09	3599
	Infrequent activity	0.81	9.7	1.09	3599
	Work/Study	0.79	9.5	1.04	3599
Male	Frequent activity	2.51	29.9	1.09	3599
	Infrequent activity	0.74	8.8	1.10	3599

Notes: Total trips are summed over the sample period; trip shares are *computed as the fraction of all trips in the country. Observations denote origin–destination–day cells (in millions). Most strata cover 3,599 districts and 306 days; smaller district counts for some Work/Study strata reflect missing observations in those groups.*

Note: Descriptive statistics for mobility flows by activity type and strata. There are fewer trips for the oldest age group working/studying, and for the highest income bracket, but it is still enough to ensure precise estimates.

2 Model description

We employ twin modeling strategies to understand the relationship between heat and mobility, the first to measure the causal effect and the second to estimate the functional form. Both assume the number of trips T terminating in district i at time t follow a Poisson distribution such that $T_{it} \sim \text{Poisson}(\mu_{it})$. Our first approach uses a two-way fixed effects model (TWFE):

$$\log(\mu_{it}) = \beta\,(\text{UTCI}_{it} \times \text{activity}) + \alpha_i + \gamma_t$$

where μ_{it} represents the expected number of trips, α_i represents district fixed effects controlling for characteristics of the *place*, while γ_t captures date fixed effects accounting for patterns common across districts at a given *time*. Here UTCI_{it} is a binary variable equal to 1 when the UTCI in district i on day t exceeds 40°C, and 0 otherwise. The interaction with activity type allows us to estimate differential temperature responses across activities. This specification leverages within-district variation in temperature after accounting for common temporal shocks, providing causal estimates under the assumption that temperature variation is as-good-as-random after controlling for location and time fixed effects. The district controls spatial confounds and the date controls are important to adjust for temporal patterns, as Spain sees activity change considerably in August as many people make holidays during this month. To implement this model, we filter out international trips to ensure our analysis captures domestic mobility patterns only. We cluster standard errors at the province level to account for spatial correlation in the error terms.

A placebo test looks for confounding variables by shuffling temperature and examining whether our model detects an effect that should not exist. Our data allow us to test both spatial and temporal confounding, and we do so by permuting the temperature either within a district, so that each district on each day is treated with temperatures from another day of the year, or within a date, so that each district on each day is treated with temperatures from another district in a different part of Spain.

To understand how heat impacts mobility across different demographic groups and to explore the functional form of the relationship, we extend our TWFE model to use binned exposure. We partition UTCI into 5°C intervals from 25–50°C and estimate:

$$\log(\mu_{it}) = \sum_b \beta_b\,\mathbf{1}\{\text{UTCI}_{it} \in \text{bin } b\} \times \text{activity} \times \text{demographic} + \alpha_i + \gamma_t,$$

where $\mathbf{1}\{\text{UTCI}_{it} \in \text{bin } b\}$ is an indicator equal to 1 when the UTCI in district i on day t falls in 5°C bin b (25–30, 30–35, 35–40, 40–45, 45–50°C), and 0 otherwise. One bin in the moderate range, with UTCI 25–30°C, serves as the omitted reference category, so the coefficients β_b are interpreted as changes in log trips relative to that baseline. UTCI is defined at the district–day level and is therefore the same for all demographic groups within a given district–day; heterogeneity arises from the interaction of these bin indicators with activity and demographic dummies. Demographic variables include age groups, income quintiles, and sex. This specification allows us to identify different responses not only across activities but also across demographic groups, providing insight into potential vulnerabilities and adaptation strategies across society. We again filter out international travel and cluster standard errors at the province level to account for spatial correlation.

Finally, we test alternative dependent variables by computing the *richness* and *evenness* of visitors to a given district. Here, instead of using T_{it} we use either richness R or evenness E. To compute

these, we assign each district a decile according to its median income; the richness is the a simple count of the number of unique income deciles that visited a district [1, 10] and evenness is proportions of those visiting deciles, using Shannon's entropy (normalized):

$$E = -\frac{\sum_{i=1}^{R} p_i \ln(p_i)}{\ln(R)}$$

where p_i represents the proportion of visitors from income decile i. Higher entropy values indicate greater diversity in the socioeconomic composition of visitors, with maximum evenness occurring when visitors are equally distributed across all present income deciles. This approach allows us to examine whether extreme heat constrains or diversifies the demographic composition of visitors to different locations, potentially revealing segregation effects that might be exacerbated by climate extremes.

Our TWFE model enables us to make causal inferences if certain conditions are met, but there are threats to the model. A key assumption is that mobility patterns would have evolved similarly across districts in the absence of temperature shocks (parallel trends). This is plausible in our setting because temperature variation is as-good-as-random after conditioning on location and time fixed effects, and districts cannot select into heat "treatment". A more serious threat to identification would be if extreme heat causes people to substitute between districts, violating the stable unit treatment value assumption (SUTVA). We test for such spillovers using a gravity model of bilateral flows between districts, explained and shown in Supplementary Table S7. After controlling for origin and destination populations and distance, we find no evidence that temperature gradients between districts drive mobility patterns, suggesting SUTVA violations are unlikely to bias our estimates.

To explore and model the potential curvilinear relationship between heat and activity, we complement the TWFE analysis with a Generalised Additive Model (GAM):

$$\log(\mu_{it}) = f_1(\mathrm{UTCI}_{it}) \times \mathrm{activity}_i + \beta_1 \mathrm{popularity}_i + \beta_2 \mathrm{province}_i + f_2(\mathrm{DoY}_t) + \mathrm{DoW}_t + \mathrm{holiday}_t$$

where $f_1(\cdot)$ is a cubic regression spline (4 knots) specified as a varying-coefficient smooth by activity type, allowing the temperature–activity relationship to differ across activities; $f_2(\cdot)$ is a cubic spline for the day-of-year, capturing seasonality and drifts in the data. We control for mean visitation (popularity) and geographic variation (province). We also add day-of-week and holiday fixed effects because, for example, weekends and holidays might have different levels of activity and this allows the intercept to vary on those days. While the TWFE isolates the causal effect, the GAM reveals the functional form of behavioural responses to temperature variation through its flexible smooth functions. The GAM's strength lies in its ability to detect and convey nonlinear relationships without imposing *a priori* assumptions about the functional form, allowing us to identify potential threshold effects and complex response patterns in human mobility.

We also employ a spatial interaction model to understand how heat specifically impacts flow dynamics between geographic areas. The gravity model, widely used in mobility studies, provides a framework for estimating the volume of flows between pairs of locations based on their attributes and the distance between them. In our application, we include temperature bands to examine how extreme heat modifies these spatial interactions. Formally, our model specification is:

$$\log(\mathrm{E}[T_{ijt}]) = \beta_1 \mathrm{UTCI}_{jt} + \beta_2(\mathrm{UTCI}_{jt} \times \mathrm{Pop}_i^q) + \beta_3(\mathrm{UTCI}_{jt} \times \mathrm{Pop}_j^q) + \beta_4 \log(d_{ij}) + \alpha_i + \gamma_j + \delta_t$$

where T_{ijt} represents the number of trips from origin i to destination j on date t, UTCI_{jt} denotes the temperature band at the destination (binned into 5°C intervals from 20-50°C), Pop_i^q and Pop_j^q are population quintiles for origin and destination districts respectively, and d_{ij} represents the distance between districts. We include origin fixed effects α_i, destination fixed effects γ_j, and date fixed effects δ_t to control for time-invariant characteristics of districts and temporal patterns. The interaction terms between temperature bands and population quintiles are particularly valuable as they allow the effect of heat to vary across the urban-rural gradient, creating different slopes for different settlement types while the fixed effects adjust the intercepts, enabling us to identify how extreme temperatures differentially impact flows between urban cores, suburban areas, and rural peripheries. This specification allows us to isolate how temperature variations modify the traditional distance decay relationship while accounting for population size—a key factor in determining trip attractiveness. We implement this as a fixed-effects Poisson model, appropriate for count data, with two-way clustering of standard errors to account for spatial correlation. This hierarchical approach enables us to decompose how extreme heat differentially affects areas along the urban-rural gradient while maintaining statistical rigor through our fixed-effects structure.

3 Model results

Table S2: Impact of Extreme Heat (UTCI $> 40°C$) on Mobility by Activity Type

**Dependent Variable:**	*Flows*	
	Within districts	Between districts
Variables		
Work or Study	-0.0068	-0.0054
	(0.0087)	(0.0079)
Frequent Activity	-0.0207**	-0.0243***
	(0.0086)	(0.0069)
Infrequent Activity	-0.125***	-0.105***
	(0.0311)	(0.0232)
Fixed effects		
District	Yes	Yes
Date	Yes	Yes
Fit statistics		
Pseudo R^2	0.978	0.972

Notes: Standard errors (in parentheses) are clustered by province.
Significance levels: *** $p < 0.01$, ** $p < 0.05$, * $p < 0.10$.

Note: Results from Poisson regressions including district and date fixed effects. Estimates represent semi-elasticities of flows (i.e., logs of expected counts). Standard errors are robust to clustering at the province level.

4 Model comparisons

Table S3: Impact of Heat (UTCI $> 30°$C) on Mobility by Age and Activity: Robustness to Excluding August in Spain

		UTCI bin (°C)			
**Activity**	**Sample**	30–35	35–40	40–45	45–50
Panel A: Age 0–25					
Frequent activity	All months	-0.0002**	-0.0145***	-0.0232***	-0.0358***
	Excluding August	0.0001	-0.0123***	-0.0232***	-0.0435***
Infrequent activity	All months	0.0114***	-0.0130***	-0.0463***	-0.0707***
	Excluding August	0.0098***	-0.0136***	-0.0495***	-0.0937***
Work/Study	All months	-0.0012***	-0.0148***	-0.0082***	-0.0207***
	Excluding August	0.0004**	-0.0114***	-0.0092***	-0.0206***
Panel B: Age 25–45					
Frequent activity	All months	-0.0004***	-0.0154***	-0.0276***	-0.0410***
	Excluding August	0.0006***	-0.0126***	-0.0297***	-0.0500***
Infrequent activity	All months	0.0101***	-0.0109***	-0.0434***	-0.0683***
	Excluding August	0.0092***	-0.0112***	-0.0437***	-0.0846***
Work/Study	All months	-0.0013***	-0.0149***	-0.0104***	-0.0222***
	Excluding August	0.0011***	-0.0110***	-0.0117***	-0.0195***
Panel C: Age 45–65					
Frequent activity	All months	-0.0008***	-0.0178***	-0.0322***	-0.0438***
	Excluding August	-0.0001	-0.0131***	-0.0325***	-0.0537***
Infrequent activity	All months	0.0101***	-0.0142***	-0.0533***	-0.0831***
	Excluding August	0.0094***	-0.0130***	-0.0543***	-0.1026***
Work/Study	All months	-0.0025***	-0.0175***	-0.0133***	-0.0251***
	Excluding August	0.0002	-0.0122***	-0.0141***	-0.0279***
Panel D: Age 65–100					
Frequent activity	All months	0.0003***	-0.0180***	-0.0355***	-0.0438***
	Excluding August	0.0011***	-0.0131***	-0.0328***	-0.0524***
Infrequent activity	All months	0.0088***	-0.0193***	-0.0591***	-0.0863***
	Excluding August	0.0092***	-0.0162***	-0.0607***	-0.1067***
Work/Study	All months	0.0003	-0.0185***	-0.0265***	-0.0345***
	Excluding August	0.0017***	-0.0136***	-0.0248***	-0.0441***

Notes: Entries are coefficients (semi-elasticities) from Poisson regressions of flows within districts on indicators for UTCI bins, interacted with age group and activity type. "All months" uses the full sample; "Excluding August" drops August observations for Spain. Regressions include district and date fixed effects; standard errors (not shown) are clustered by province. Pseudo R^2 across these models is 0.972 (All months) and 0.967 (Excluding August). Significance levels: *** $p < 0.01$, ** $p < 0.05$, * $p < 0.10$.

Table S4: Impact of Heat (UTCI $> 30°$C) on Mobility by Income and Activity: Robustness to Excluding August in Spain

**Activity**	**Sample**	*UTCI bin (°C)* 30–35	35–40	40–45	45–50
Panel A: Income < 10					
Frequent activity	All months	0.0124***	0.0300***	0.0295***	0.0202***
	Excluding August	0.0134***	0.0352***	0.0379***	0.0335***
Infrequent activity	All months	0.0312***	0.0311***	-0.0139***	-0.0295***
	Excluding August	0.0263***	0.0263***	-0.0236***	-0.0502***
Work/Study	All months	0.0096***	0.0220***	0.0305***	0.0260***
	Excluding August	0.0095***	0.0279***	0.0390***	0.0418***
Panel B: Income 10–15					
Frequent activity	All months	0.0008***	-0.0177***	-0.0391***	-0.0480***
	Excluding August	0.0003***	-0.0158***	-0.0385***	-0.0587***
Infrequent activity	All months	0.0122***	-0.0124***	-0.0573***	-0.0873***
	Excluding August	0.0098***	-0.0155***	-0.0623***	-0.1127***
Work/Study	All months	-0.0001*	-0.0178***	-0.0251***	-0.0324***
	Excluding August	0.0011***	-0.0132***	-0.0214***	-0.0323***
Panel C: Income > 15					
Frequent activity	All months	-0.0028***	-0.0255***	-0.0525***	-0.0997***
	Excluding August	-0.0057***	-0.0264***	-0.0549***	-0.1131***
Infrequent activity	All months	0.0014***	-0.0296***	-0.0575***	-0.1326***
	Excluding August	-0.0002	-0.0250***	-0.0453***	-0.1351***
Work/Study	All months	-0.0010***	-0.0188***	-0.0252***	-0.0590***
	Excluding August	-0.0012***	-0.0204***	-0.0308***	-0.0700***

Notes: Entries are coefficients (semi-elasticities) from Poisson regressions of flows within districts on indicators for UTCI bins, interacted with income group and activity type. "All months" uses the full sample; "Excluding August" drops August observations for Spain. Regressions include district and date fixed effects; standard errors (not shown) are clustered by province. Pseudo R^2 across these models is 0.9705 (All months) and 0.9704 (Excluding August). Significance levels: *** $p < 0.01$, ** $p < 0.05$, * $p < 0.10$.

Table S5: Impact of Heat (UTCI $> 30°$C) on Mobility by Gender and Activity: Robustness to Excluding August in Spain

		UTCI bin (°C)			
**Activity**	**Sample**	30–35	35–40	40–45	45–50
Panel A: Female					
Frequent activity	All months	0.0004***	-0.0174***	-0.0290***	-0.0451***
	Excluding August	0.0006***	-0.0126***	-0.0289***	-0.0512***
Infrequent activity	All months	0.0093***	-0.0156***	-0.0491***	-0.0799***
	Excluding August	0.0085***	-0.0144***	-0.0486***	-0.0967***
Work/Study	All months	-0.0012***	-0.0167***	-0.0133***	-0.0261***
	Excluding August	0.0007***	-0.0114***	-0.0130***	-0.0223***
Panel B: Male					
Frequent activity	All months	0.0004***	-0.0168***	-0.0298***	-0.0440***
	Excluding August	0.0005***	-0.0128***	-0.0305***	-0.0538***
Infrequent activity	All months	0.0104***	-0.0150***	-0.0504***	-0.0799***
	Excluding August	0.0096***	-0.0142***	-0.0508***	-0.0999***
Work/Study	All months	-0.0014***	-0.0164***	-0.0121***	-0.0286***
	Excluding August	-0.0001	-0.0123***	-0.0128***	-0.0345***

Notes: Entries are coefficients (semi-elasticities) from Poisson regressions of flows within districts on indicators for UTCI bins, interacted with gender and activity type. "All months" uses the full sample; "Excluding August" drops August observations for Spain. Regressions include district and date fixed effects; standard errors (not shown) are clustered by province. Pseudo R^2 across these models is 0.980 (All months) and 0.982 (Excluding August). Significance levels: *** $p < 0.01$, ** $p < 0.05$, * $p < 0.10$.

Table S6: Impact of Heat on Network Mixing (Richness and Evenness):
Robustness to Excluding August in Spain

		UTCI bin (°C)			
**Metric**	**Sample**	30–35	35–40	40–45	45–50
Panel A: Population-based mixing					
Evenness	All months	0.00019	-0.00059	-0.00201**	-0.00267**
	Excluding August	0.00009	-0.00028	-0.00143**	-0.00209
Richness	All months	0.0173***	0.0218**	-0.0154	-0.0541*
	Excluding August	0.0167***	0.0150	-0.0162	-0.0680
Panel B: Income-based mixing					
Evenness	All months	0.00019	-0.00091	-0.00237**	-0.00110
	Excluding August	0.00008	-0.00029	-0.00085	0.00160
Richness	All months	0.0227***	0.0212	-0.0749***	-0.1613***
	Excluding August	0.0194***	0.0201	-0.0943***	-0.1543**

Notes: Entries are coefficients from OLS regressions of district-day mixing metrics (richness or evenness) on indicators for UTCI bins, with the 25–30°C bin omitted as the reference category. Coefficients are reported in the original units of the mixing metrics (richness/evenness); for interpretability, the Fig. 5 in the main text presents the same estimates rescaled to percent change relative to the reference bin. "All months" uses the full sample; "Excluding August" drops August observations for Spain. Regressions include district and date fixed effects; standard errors (not shown) are clustered by province. Significance levels: *** $p < 0.01$, ** $p < 0.05$, * $p < 0.10$.

5 Sensitivity analysis

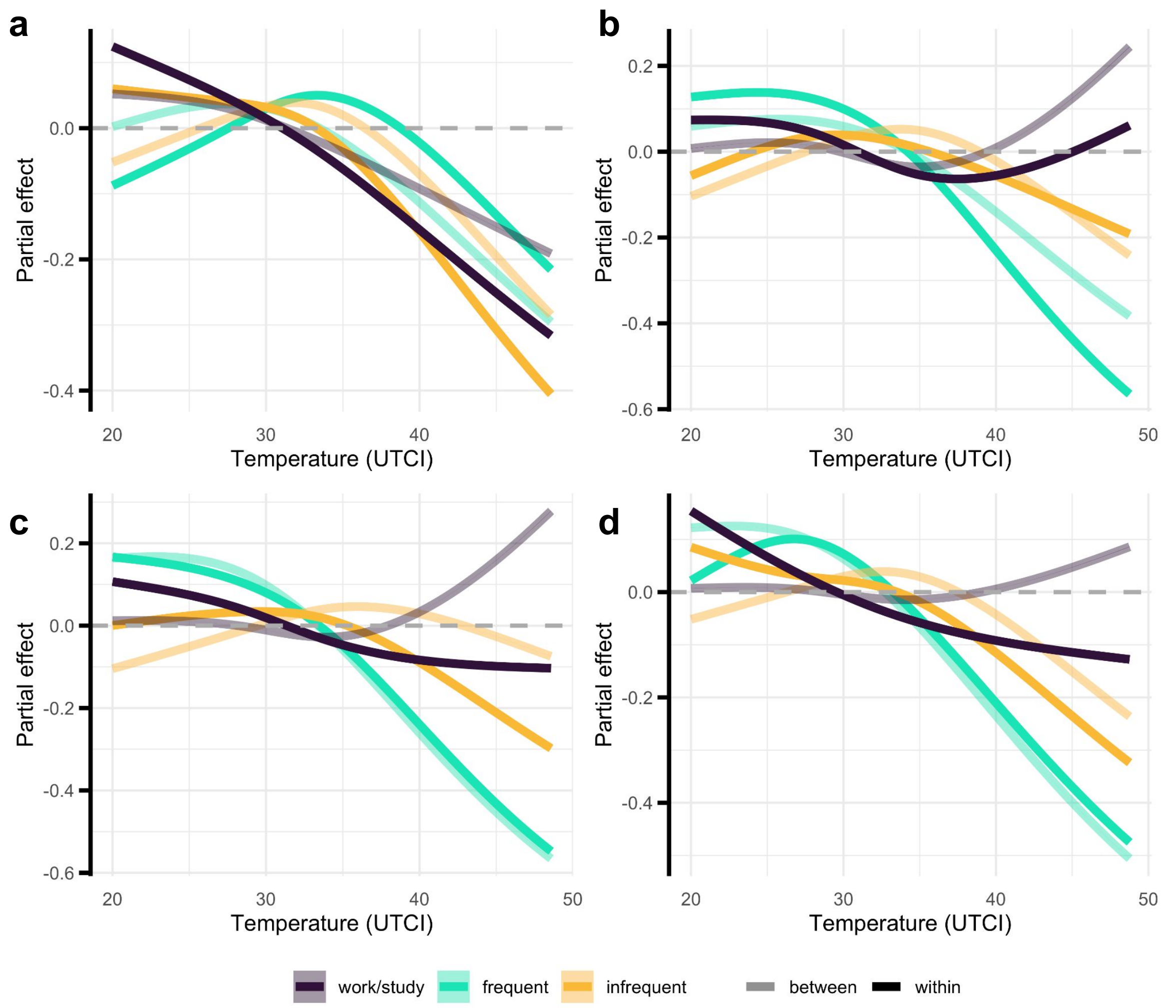

Figure S4: Sensitivity analysis. **A** Using the same GAM model specified in the Methods section, with flows into a district as the dependent variable, results for the top 50 cities in Spain, all with a population over 100,000, and **B** for the remainder of Spanish cities, showing that the results are largely stable, with the possible exception being around work patterns: in large cities, people are more likely to avoid travel to work on hot days than in small cities—interesting because information technology jobs concentrate in large cities and thus can likely be taken from home [8]. Curves show partial effects for the same activity types as in the main text; e.g. yellow line corresponds to *infrequent* activities, as indicated in the legend. **C** and **D** use the same model for all districts in Spain, but restrict the data to 2022 and 2023, respectively, showing that the estimated patterns are also stable across time.

6 Placebo tests

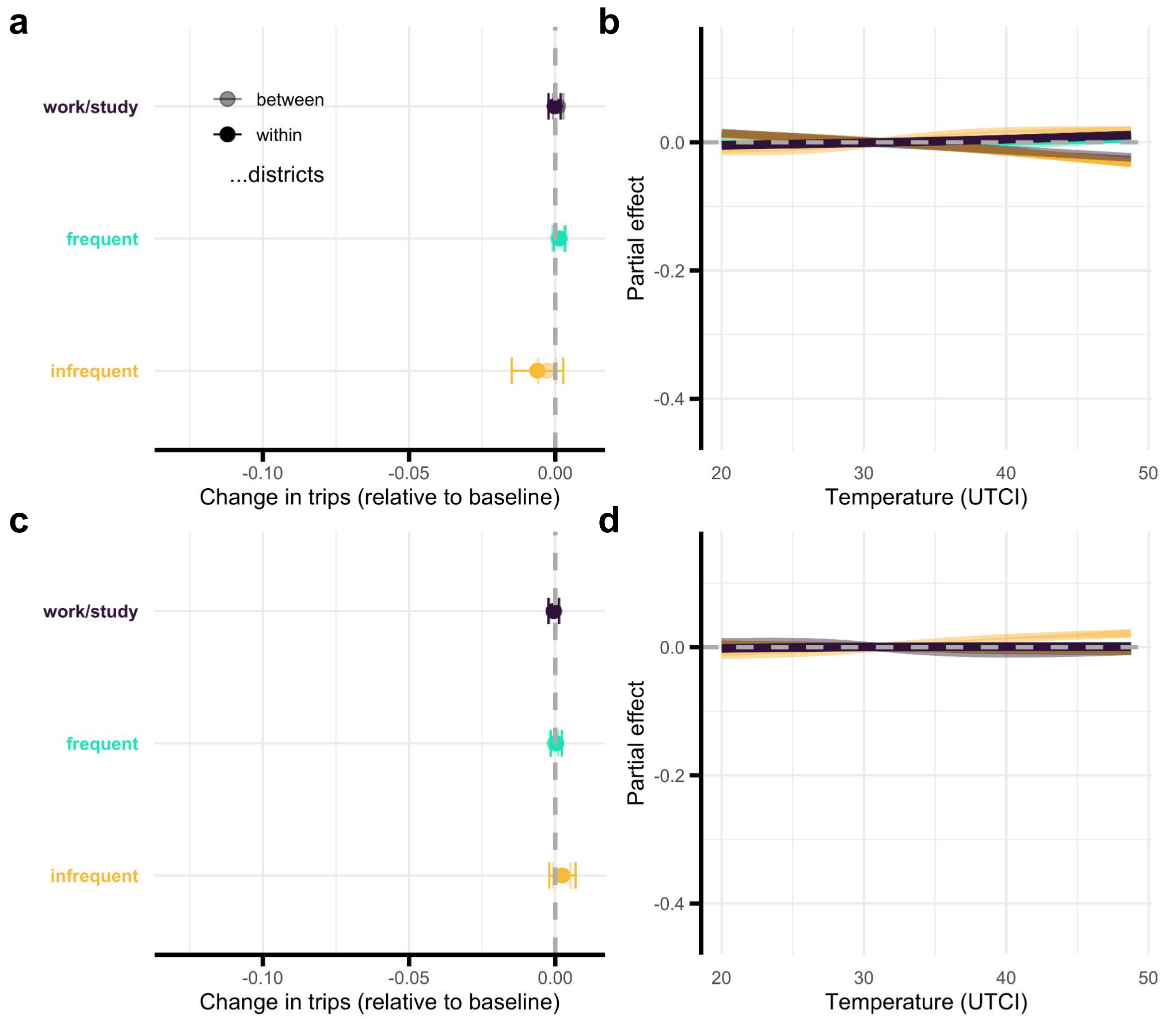

Figure S5: Placebo tests. We perform 4 separate placebo tests to rule out potential spurious associations: **A** and **B** shuffle temperatures across districts on the same date, while **C** and **D** shuffle temperatures across different dates within the same district. The cross-district shuffles help rule out the possibility that our results are driven by events affecting all districts simultaneously. The within-district shuffles help rule out the possibility that observed changes would occur within districts regardless of temperature variation. While GAM confidence intervals occasionally exclude zero, effect sizes are reduced by an order of magnitude or more compared to the main analysis, supporting our primary findings.

7 Consistency between groups

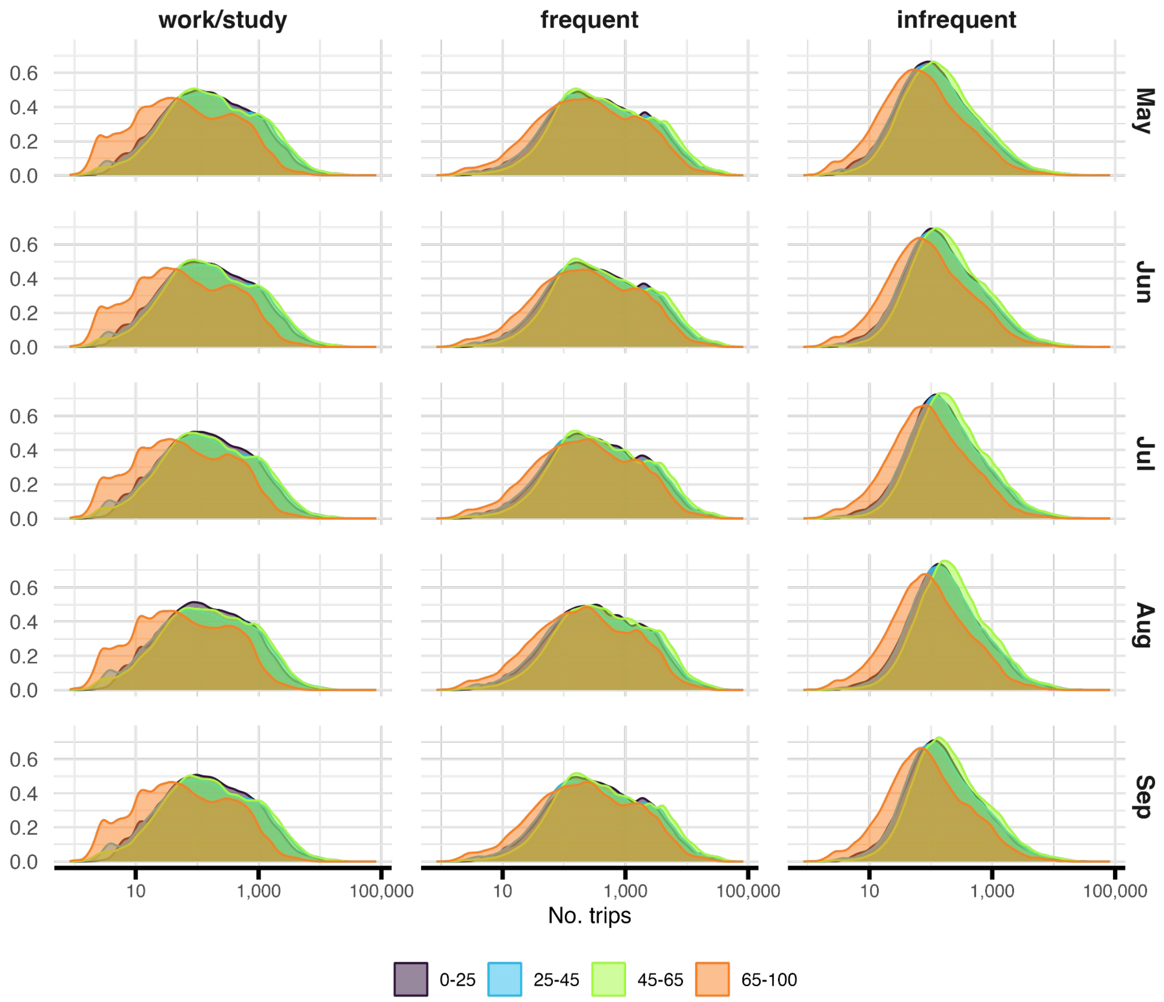

Figure S6: Trip distributions between ages. Here we show the distributions of daily visits per month and class of trip. We note that across all months and classes of trip, the distributions of trips are generally consistent between ages, with fewer trips amongst the oldest age bracket.

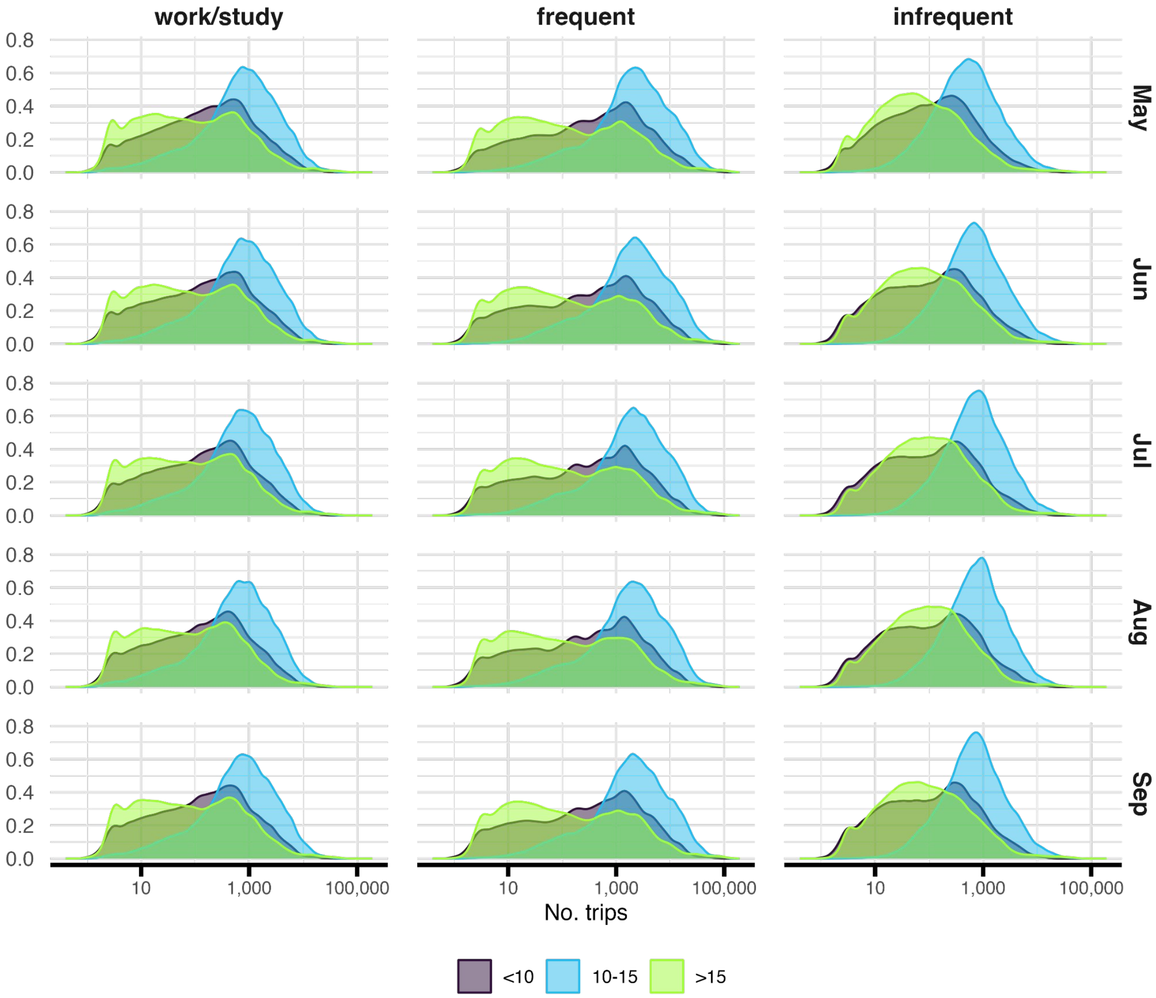

Figure S7: Trip distributions between income. Here we show the distributions of daily visits per month and class of trip. We note that across all months and classes of trip the most prevalent group in the data is the middle class.

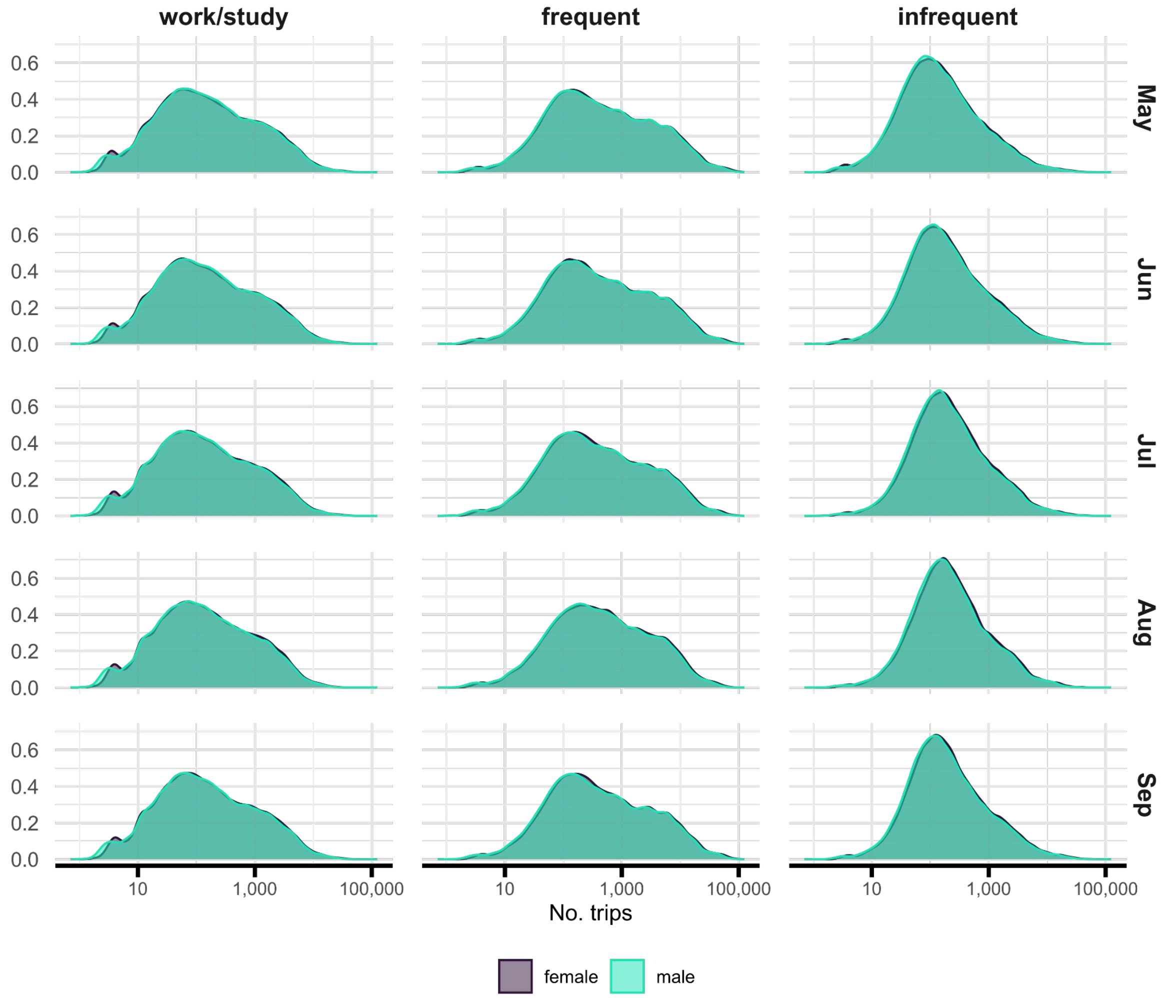

Figure S8: Trip distributions between genders. Here we show the distributions of daily visits per month and class of trip. We note that across all months and classes of trip, the distributions of trips are consistent between genders, suggesting broad similarity.

8 Gravity model

Dependent Variable:		Trips	
	Base model	Temperature bins	With gradient
Model:	(1)	(2)	(3)
Variables			
Log Distance	-1.87***	-1.87***	-1.87***
	(0.013)	(0.013)	(0.013)
Temperature 25-30°C		0.011***	0.012***
		(0.002)	(0.002)
Temperature 30-35°C		0.008**	0.009***
		(0.004)	(0.004)
Temperature 35-40°C		-0.020***	-0.018***
		(0.006)	(0.006)
Temperature 40-45°C		-0.053***	-0.051***
		(0.007)	(0.007)
Temperature 45-50°C		-0.066***	-0.062***
		(0.009)	(0.010)
Log Distance × Temperature Gradient			-0.001*
			(0.0007)
Fixed-effects			
ID origin	Yes	Yes	Yes
ID destination	Yes	Yes	Yes
date	Yes	Yes	Yes
Fit statistics			
Observations	31,148,196	31,148,196	31,148,196
Pseudo R^2	0.87382	0.87386	0.87386
Squared Correlation	0.78625	0.78636	0.78637

Clustered (origin & destination) standard-errors in parentheses
*Signif. Codes: ***: 0.01, **: 0.05, *: 0.1*

Table S7: Impact of temperature on mobility. We use gravity models to understand if flows are displaced, moving from one district to another, during heat waves, which would violate TWFE assumptions. While high temperatures reduce flows, agreeing with the TWFE analysis, we see that temperature gradient has no significant effect, suggesting that people do not preference higher or lower temperatures during heat waves. This also indicates that people do not seek out cooler parts of the country during extreme heat. All models include origin, destination, and date fixed effects; Standard errors clustered by origin and destination.

	Population				
Temperature	Very Low	Low	Medium	High	Very High
25-30°C	0.0267***	0.0324***	0.0321***	0.0376***	0.0482***
	(0.0031)	(0.0045)	(0.0086)	(0.0091)	(0.0092)
30-35°C	0.0589***	0.0629***	0.0371***	0.0277**	0.0224**
	(0.0049)	(0.0059)	(0.0078)	(0.0080)	(0.0084)
35-40°C	0.0862***	0.0834***	0.0350***	-0.0007	-0.0363***
	(0.0075)	(0.0095)	(0.0077)	(0.0076)	(0.0077)
40-45°C	0.0791***	0.0763***	0.0086	-0.0303**	-0.0874***
	(0.0081)	(0.0108)	(0.0083)	(0.0089)	(0.0087)
45-50°C	0.0751***	0.0588***	0.0123	-0.0783***	-0.0962***
	(0.0158)	(0.0125)	(0.0130)	(0.0173)	(0.0146)
log(d)			-1.865*** (0.0112)		
Fixed-effects					
ID origin			Yes		
ID destination			Yes		
date			Yes		
Fit statistics					
Observations			19,638,009		
Pseudo R^2			0.87786		
Squared Correlation			0.79586		

Clustered (by origin & destination) standard-errors in parentheses
*Signif. Codes: ***: 0.001, **: 0.01, *: 0.05, .: 0.1*

Table S8: Impact of temperature and combined population on mobility. In this gravity model we report the results of a gravity model that groups flows by population at origin and destination. Because the model has 5x5 population classes as well as temperature bands, we present the average effects given population group at destination. Our results show large negative effects for the most populous areas, while moderate warmth is positive relative to cold.

9 Projecting into the future

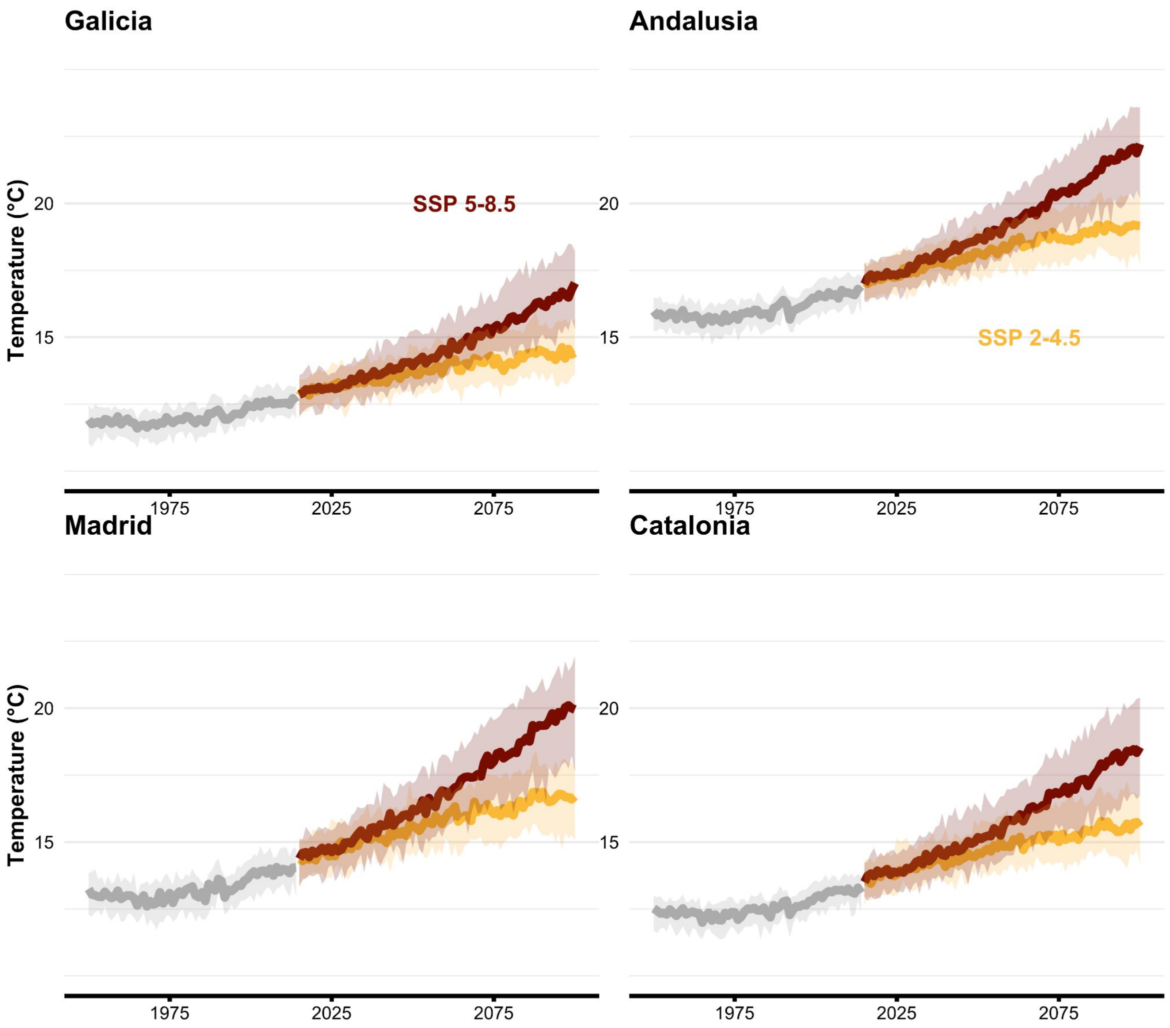

Figure S9: CMIP estimates. Andalusia, in the South will see annual temperatures rise more than Galicia, in the North. Major cities like Barcelona and Madrid will also experience considerable changes.

We use modeled estimates of Earth's future climate, derived from CMIP6 [9], which represents the state-of-the-art in climate projections. (For context, Fig. S9 shows projections for major cities.) We extract data on temperature, humidity, radiation, and wind to compute UTCI manually for the year 2073 under the $2 - 4.5$°C of warming, using the simulation from the Centre National de Recherches Météorologiques in France, because it shows high accuracy against observed data [10] and of comparably accurate models it performed best in Spain when we checked it against our data for 2022 and 2023. The strategy that we employ here is simple: we switch out the UTCI for a given district and day in 2023 for the UTCI on that day in 2073. Because the GAM allows us to produce estimates across the full range of temperatures, we make our predictions using this model rather than the TWFE. We compare predicted values using 2023 temperatures to predicted values using 2073 data to ensure that

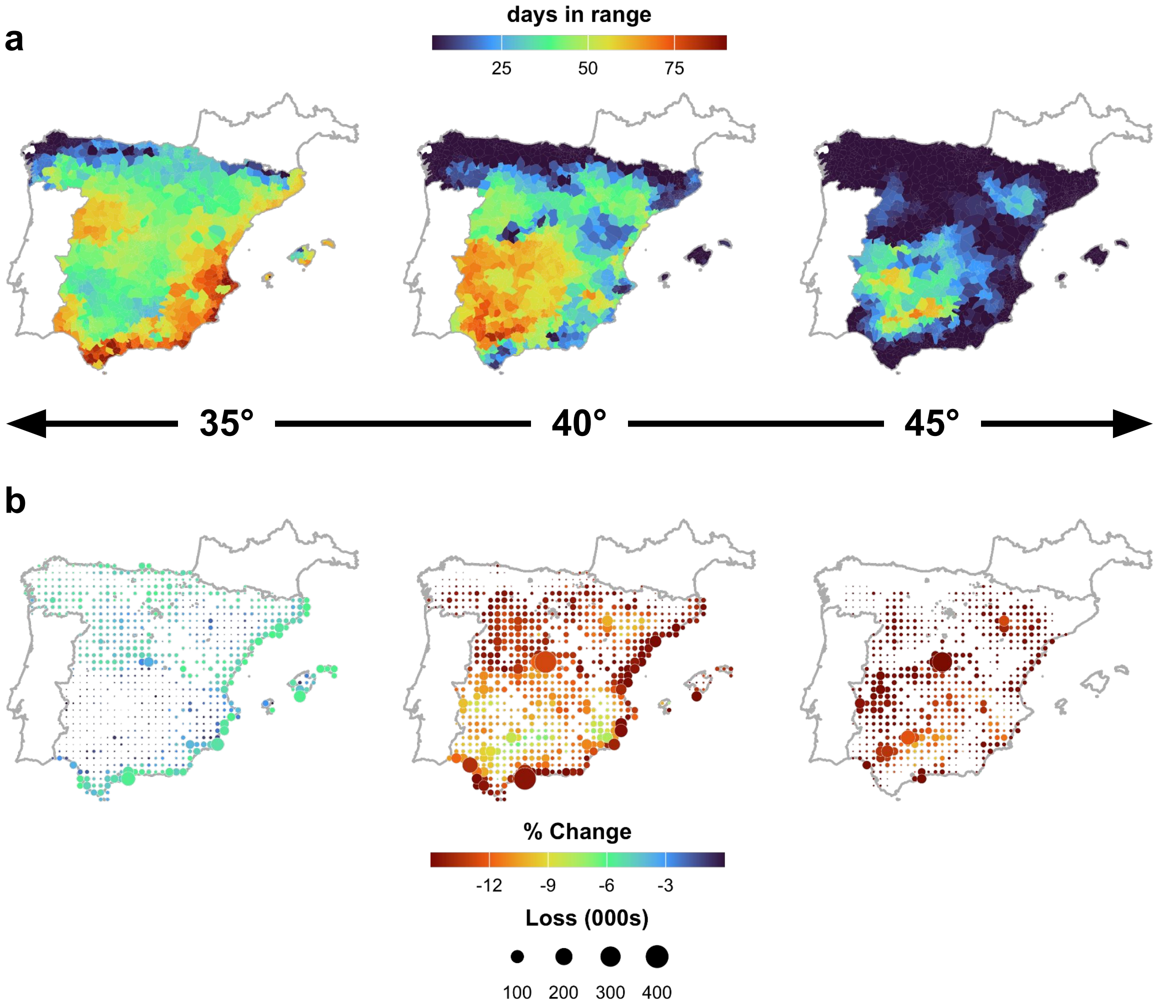

Figure S10: Projecting in the future. **A** Prevalence of temperature exceeding a given value. Maps show that Spain's South will see 75 days between 40°C and 45°C as well as dozens of days above 45°C; days between 35°C and 40°C will also be more common in the North. **B** Consequences of those temperatures in both relative and absolute terms per district, showing that according to our model the largest effects at lower temperatures will be in areas that do not experience high temperatures now. Cities in the South like Sevilla and Malaga will experienced the highest temperatures, however, they do not see the strongest drop in relative terms; big cities like Madrid and Barcelona will see the largest change in absolute terms.

we are comparing like with like, modeled estimates in both cases, rather than using observed values in one and predicted values in another.

Our estimates assume that temperature and nothing else changes going forward. Although the "Lucas Critique" [11] tells us to be wary of making projections when humans can adapt to changing circumstances [12], Spain is ageing and thus the demographic issues that we highlight above could be exacerbated. The exercise is also informative because activity is fundamentally different to mortality and morbidity, where we do see evidence for adaptation in recent years [13, 14]: travel within and

between cities will likely require, for the foreseeable future, contact with the ambient air. Coping with heat may reduce mobility more, not less, as we attempt to reduce its worst effects on health. For example, it may become more common for employees to work from home on hot days, as telework has changed the demand for face-to-face interaction [15]. For this reason, we believe our estimates to have important implications for the future economy of cities and towns, including, for example, urban business that depend on office workers or rural areas that depend on tourists [16].

Our model predicts a 3.5% reduction in frequent activities during summer months, May to September, and a 4.7% reduction in infrequent activities. Travel to work or school is projected to fall by 2.2% during that same period. Yet over the full year, all activities will decline by just 1%, as warmer weather during what were once cold periods increases mobility while hotter weather during already hot seasons decreases it.

Next we explore how this could vary across cities and regions. Fig. S10**A** shows the number of days spent in a given UTCI range, from $35-40$°C to $40-45$°C to $45-50$°C. All of these temperatures correspond to reductions in activity according to our models, and they will be common in the South of the country. In particular, parts of Andalusia will see temperatures exceed 40°C for more than 75 days each year. The North of Spain will experience more days that exceed 35°C, but will only have a few days a year at the extremes that the South will endure.

Fig. S10**B** shows that the effects of these changes may change mobility if adaptations do not address rising temperatures. Our estimates here show the change on a given day at a given temperature, compared to the baseline. Effect sizes are larger at lower temperatures in the North, where society is not as well acclimated to heat. Because extremes that will occur in the South will not be prevalent there, we concentrate on temperatures between 35°C and 40°C; days in this range would still reduce activity by as much as 6% on days when they occur. While the South is largely unaffected by these temperatures, it see strong changes to activity when temperatures exceed 40°C; when this occurs, activity in the South will fall by 6-12% while activity in the remainder of the country could fall be 15%.

References

[1] Ministry of Transport, Mobility and Urban Agenda of Spain, MITMA. Open Data Movilidad 2020-2021. https://www.transportes.gob.es/ministerio/proyectos-singulares/estudios-de-movilidad-con-big-data/opendata-movilidad (2024).

[2] Kotov, E., Lovelace, R. & Vidal-Tortosa, E. *spanishoddata* (2024). URL https://github.com/rOpenSpain/spanishoddata.

[3] Moro, E., Calacci, D., Dong, X. & Pentland, A. Mobility patterns are associated with experienced income segregation in large us cities. *Nature communications* **12**, 4633 (2021).

[4] de la Prada, À. G. & Small, M. L. How people are exposed to neighborhoods racially different from their own. *Proceedings of the National Academy of Sciences* **121**, e2401661121 (2024).

[5] Secretaría de Estado de Transportes y Movilidad Sostenible. Estudio de movilidad de viajeros de ámbito nacional aplicando la tecnología Big Data: Informe metodológico. Technical Report, Ministerio de Transportes y Movilidad Sostenible (2024).

[6] Subdirección General de Planificación, Red Transeuropea y Logística. Controles de calidad realizados a los análisis de movilidad con BigData. Parte 1. Estudios básicos. Technical Report, Ministerio de Transportes y Movilidad Sostenible (2024).

[7] Di Napoli, C., Barnard, C., Prudhomme, C., Cloke, H. L. & Pappenberger, F. Era5-heat: a global gridded historical dataset of human thermal comfort indices from climate reanalysis. *Geoscience data journal* **8**, 2–10 (2021).

[8] EURopean Employment Services. Labour market information: Spain (2024). URL https://eures.europa.eu/living-and-working/labour-market-information-europe/labour-market-information-spain_en. Accessed: December 12, 2024.

[9] Thrasher, B. *et al.* Nasa global daily downscaled projections, cmip6. *Scientific data* **9**, 262 (2022).

[10] Craigmile, P. F. & Guttorp, P. Comparing cmip6 climate model simulations of annual global mean temperatures to a new combined data product. *Earth and Space Science* **10**, e2022EA002468 (2023).

[11] Lucas Jr, R. E. Econometric policy evaluation: A critique. In *Carnegie-Rochester conference series on public policy*, vol. 1, 19–46 (North-Holland, 1976).

[12] Kahn, M. E. *Adapting to climate change* (Yale University Press, 2021).

[13] Heutel, G., Miller, N. H. & Molitor, D. Adaptation and the mortality effects of temperature across us climate regions. *Review of Economics and Statistics* **103**, 740–753 (2021).

[14] Barreca, A., Clay, K., Deschenes, O., Greenstone, M. & Shapiro, J. S. Adapting to climate change: The remarkable decline in the us temperature-mortality relationship over the twentieth century. *Journal of Political Economy* **124**, 105–159 (2016).

[15] Barrero, J. M., Bloom, N. & Davis, S. J. The evolution of work from home. Tech. Rep., National Bureau of Economic Research (2023).

[16] Yabe, T., García Bulle Bueno, B., Frank, M. R., Pentland, A. & Moro, E. Behaviour-based dependency networks between places shape urban economic resilience. *Nature human behaviour* 1–11 (2024).
